# Nature-Based Interventions for Individuals with Psychiatric Disorders: A Mixed Methods Systematic Review with Random-Effects Meta-Analysis of Mental Health and Functional Outcomes

**DOI:** 10.3390/bs16060974

**Published:** 2026-06-11

**Authors:** Alessandra Giammanco, Erin Grace Lawrence, Ailbhe Madigan, Karol Basta, Giada Tripoli, Aisling O’Neill, Natasha Moses, Helena Farstad, Peter Coventry, Uzma Zahid

**Affiliations:** 1 Department of Biomedicine, Neuroscience and Advanced Diagnostics, University of Palermo, 90129 Palermo, Italy; alessandra.giammanco02@unipa.it (A.G.); giada.tripoli@unipa.it (G.T.); 2 Department of Medical Sciences and Public Health, University of Cagliari, 09042 Monserrato, Italy; 3 Centre for Psychiatry and Mental Health, Wolfson Institute of Population Health, Queen Mary University of London, London E1 2AB, UK; e.g.lawrence@qmul.ac.uk; 4 Institute of Psychiatry, Psychology & Neuroscience, King’s College, London SE5 8AF, UK; ailbhe.madigan.25@ucl.ac.uk; 5 Public Health, Royal Free Hospital, London NW3 2QG, UK; karol.basta1@nhs.net; 6 Department of Psychology, St. Patrick’s University Hospital, D08 K7YW Dublin, Ireland; aisoneill@stpatricks.ie; 7 South West London and St. George’s Mental Health NHS Trust, London SW17 0YF, UK; 8 Independent Researcher, London SE5 8AF, UK; helenafmckeown@gmail.com; 9 School of Nursing and Public Health, Manchester Metropolitan University, Manchester M15 6GX, UK; p.coventry@mmu.ac.uk

**Keywords:** nature-based interventions, psychosis, schizophrenia, mental health, wellbeing

## Abstract

Nature-based interventions (NBIs) are increasingly used in mental health services, but their effectiveness in people with psychiatric disorders, and how these individuals experience them, remains unclear. This review synthesised quantitative and qualitative evidence on NBIs in psychiatric populations. Eligible studies evaluated outdoor NBIs against controlled comparators, excluding neurodevelopmental/degenerative conditions and indoor or virtual interventions. Quantitative outcomes were synthesised using random-effects meta-analysis; qualitative data were analysed using thematic synthesis. Twenty-eight studies were included, mostly involving people with diagnoses of schizophrenia or depression. NBIs were associated with greater improvements in clinical symptoms than controlled comparators (pooled effect size 0.71 [95% CI 0.29–1.12]; *p* = 0.0009), with moderate heterogeneity (I^2^ = 48.6%). The qualitative synthesis identified five themes: Being in Nature, Personal Growth, Psychological Wellbeing, Social Relationships, and Physical Benefits. Participants reported reduced stress, improved mood and coping, strengthened identity, enhanced social connection, and increased energy. NBIs, particularly horticultural programmes and guided outdoor activities, may offer promising recovery-oriented adjuncts to psychiatric care. The next step is to build a translational evidence base by harmonising recovery-relevant outcomes and developing pragmatic, scalable models of delivery that can be embedded within routine mental health services, informed by mixed methods evaluation.

## 1. Introduction

Psychiatric disorders are among the leading causes of disability worldwide, contributing substantially to years lived with disability and overall disease burden (Murray, 2022). Recent estimates suggest that one in eight people globally are affected by a mental health condition (GBD 2019 Mental Disorders Collaborators, 2022). In addition to premature mortality (Adorjan & Falkai, 2019) and high relapse rates (Solmi et al., 2023), psychiatric disorders are associated with functional impairment (Alonso et al., 2011), social exclusion (Reinhard et al., 2020), and economic costs for individuals, families, and healthcare systems (Trautmann et al., 2016). Despite advances in pharmacotherapy and psychological treatments, approximately 40–60% of people with major depressive disorder, bipolar disorder, or schizophrenia do not sustain recovery over 1–2 years, and ≥60% relapse or recur over 5–10 years (Radua et al., 2017; Hardeveld et al., 2010; Jääskeläinen et al., 2013). This underscores the need for complementary approaches to psychiatric rehabilitation.

Growing availability via commissioning and prescribing pathways in nature-based interventions (NBIs) (Fullam et al., 2021) reflects recognition that structured activities in natural environments—such as horticultural therapy, green exercise, care farming, and forest therapy—can complement standard psychiatric treatments and increase the chances of sustained recovery over time (Shanahan et al., 2019). The rationale for NBIs is grounded in several overlapping theoretical perspectives. Evolutionary and biophilia-based accounts propose that humans have an innate tendency to attend to and derive benefit from natural environments, reflecting the adaptive significance of nature across human development. Broader ecological conceptualisations of psychological health similarly emphasise that mental health is shaped not only by individual symptoms or treatments, but also by the quality of a person’s relationships with their social, physical, and environmental contexts.

NBIs are thought to act through mutually reinforcing pathways. The psychological mechanisms include stress reduction (Johansson et al., 2022), improved emotion regulation, restoration of attention (Kaplan, 1995), increased physical activity, opportunities for social connection (Coventry et al., 2021), and the development of psychological connection with nature. This latter pathway is distinct from nature contact alone, participants may benefit not only from being in natural environments, but from developing a sense of belonging, meaning, identity, and emotional attachment in relation to nature. These processes align closely with recovery-oriented models of care, which emphasise autonomy, identity, connectedness, hope, and quality of life.

There are also plausible neurobiological pathways through which outdoor NBIs may support psychiatric rehabilitation exposure to natural daylight, particularly in the morning, may strengthen circadian entrainment and stabilise sleep–wake rhythms, which are frequently disrupted in depression, bipolar disorder, and psychosis and are increasingly recognised as relevant to psychiatric pathophysiology and treatment (Monteleone et al., 2011). Outdoor activity may also provide regular behavioural timing cues through movement, routine, and social engagement, further supporting circadian regulation and sleep. In parallel, NBIs may influence HPA-axis and stress-regulatory systems, given evidence that chronic psychiatric disorders, including schizophrenia, can be associated with altered cortisol dynamics and stress responsivity (e.g., flattened cortisol awakening responses) (Aas et al., 2019). Together, these pathways suggest that NBIs may plausibly affect both psychological recovery processes and biological systems implicated in mental health.

Evidence from general populations suggests that NBIs can improve mood (McMahan & Estes, 2015), reduce stress (Twohig-Bennett & Jones, 2018), and enhance wellbeing (Shanahan et al., 2019), and they are increasingly promoted in public health and social policy as acceptable, community-based approaches (Bickerdike et al., 2017), with emerging evidence of cost-effectiveness in some settings (Hinde et al., 2021; Busk et al., 2022). Their use in psychiatric populations remains underexplored, despite strong theoretical alignment with recovery-oriented models of care, which emphasise autonomy, identity, social inclusion, and quality of life.

While interest in NBIs has surged, the evidence base has remained heterogeneous. Previous reviews have largely focused on general populations (Coventry et al., 2021; Nguyen et al., 2023; Twohig-Bennett & Jones, 2018) or specific conditions (Jessen et al., 2025), often using narrative (Harrison et al., 2023) or scoping approaches (Wilkie & Davinson, 2021), and recently published umbrella reviews have begun synthesising this growing body of evidence at a high level (Brandt et al., 2026; Shrestha et al., 2025; Kaleta et al., 2025). However, few reviews have specifically examined outcomes in people with diagnosed psychiatric disorders—including anxiety (Jessen et al., 2025), depression (Jessen et al., 2025; Salomon et al., 2018), and stress-related conditions (Jessen et al., 2025; Paredes-Céspedes et al., 2024)—and those that do have focused almost exclusively on quantitative efficacy, with comparatively limited attention to participant experiences, implementation processes, and the meaning participants ascribe to these interventions (Cuthbert et al., 2021). No review to date has integrated quantitative and qualitative evidence in this population.

We aimed to address these gaps by systematically reviewing and meta-analysing studies of NBIs for individuals with psychiatric disorders, encompassing a broad spectrum of diagnoses and intervention types, while integrating quantitative effect sizes with qualitative thematic synthesis, allowing us to identify not only if NBIs are effective across clinical and functional domains, but also how individuals with psychiatric lived experience perceive, value, and engage with these interventions.

## 2. Materials and Methods

### 2.1. Search Strategy and Study Selection

The systematic review protocol was registered with PROSPERO (CRD42024581439) and reported in accordance with PRISMA guidelines (Moher et al., 2009). Ovid Embase; Ovid Medline; Ovid PsycINFO; Global Health; Scopus; Green File via EBSCOhost; Web of Science; and the Cochrane Central Register of Controlled Trials were searched on 4 October 2024 with no limits for language, study design, or publication date. The search strategies used text words and exploded index terms to retrieve relevant literature. Full database-by-database search strategies are provided in the Supplementary Materials (File S1 pp. 2–18).

All references were exported to Rayyan (Ouzzani et al., 2016) where automated duplication was run followed by manual removal. Titles and abstracts were screened independently by five reviewers (AM, UZ, GT, AG, EGL). Full-text screening was subsequently conducted in duplicate by pairs of reviewers. At both stages, disagreements were resolved through discussion until consensus was reached.

Studies were eligible for inclusion if they involved participants with psychiatric disorders (i.e., mood disorders, anxiety disorders, schizophrenia spectrum, bipolar disorder, PTSD, personality disorders) in any mental health or psychiatric rehabilitation setting, excluding those with primary neurodevelopmental or neurodegenerative conditions. We included studies conducted in mental health or psychiatric rehabilitation contexts, including interventions delivered off-site in non-clinical green spaces (e.g., care farms, community gardens, parks, or forests) provided the intervention was accessed via, delivered within, or linked to a mental health or rehabilitation pathway. Interventions had to be outdoor nature-based activities (e.g., green-space, farm-based, or animal-assisted programmes) and were excluded if delivered indoors only, in non-natural settings, or via virtual/simulated nature. Eligible comparators included treatment as usual, non-nature-based activities or therapies, waitlist, or no intervention.

### 2.2. Data Extraction

Outcome domains were defined a priori during protocol development and informed by the PICO framework. These were used as guiding categories for outcome extraction and synthesis. Specifically, “Clinical Symptoms” referred to psychiatric symptom severity, including depression, anxiety, stress, and psychotic symptoms. “Quality of Life and Wellbeing” outcomes included individual evaluations of life satisfaction, mental wellbeing, self-compassion, and perceived quality of life. “Psychosocial” outcomes referred to interpersonal and self-related constructs such as self-efficacy, coping strategies, self-esteem, and social participation. “Functional outcomes” captured engagement in daily and meaningful activities and social functioning. Last, “Physical Health” outcomes reported physical health benefits.

One author (either AG, EGL, UZ) independently extracted the study characteristic data into a structured template. Data were second checked by two authors (EGL and KB). Any discrepancies were discussed between authors, and unresolved issues were reconciled with a senior researcher (UZ).

Extracted data for all studies covered general study characteristics, (bibliographic details such as title, year, region; methodological design; definition and theme of NBI; and psychiatric setting,), participant characteristics (psychiatric diagnosis, diagnostic criteria, comorbidities, sample size, age, gender, ethnicity) and intervention and control group characteristics (intervention content, duration, frequency, timepoints, assessment timing, and control condition details). For studies with quantitative outcomes (including mixed methods studies), data were extracted and organised into the following domains: clinical symptoms, functioning, quality of life and wellbeing, psychosocial outcomes, and physical health. For each outcome, we recorded the outcome measurement tool, subscale, group, statistical test, pre- and post-intervention means (SD), and *p* values.

Extracted data (from qualitative and mixed methods studies) covered data collection methods, analysis technique, theoretical underpinning, and thematic findings. Additionally, all text labelled as “results” or “findings” were extracted verbatim (Thomas & Harden, 2008), and input into NVIVO (version 15.2.0) for analysis.

### 2.3. Study Quality

Given the mixed methods nature of this review and the inclusion of studies with diverse designs, the methodological quality of all included studies was assessed using the Critical Appraisal Skills Programme (CASP) checklists for Randomised Controlled Trials, Cohort Studies, and Qualitative Research (Long et al., 2020).

Two authors (AG and NM) conducted assessments independently, with disagreements resolved through discussion and, if necessary, adjudication by with a third researcher (EGL). For each study, items answered “Yes” were scored as 1 and items answered “No” or “Can’t Tell” as 0, and the proportion of applicable items fulfilled was calculated. Thresholds for classification were defined a priori as follows: low = <60% of items fulfilled; moderate = 60–79%; high = ≥80%. The two reviewers achieved an agreement rate of 95%.

### 2.4. Random-Effects Meta-Analysis

Quantitative analyses were conducted in Stata/MP 19.0 for Mac (Apple Silicon) using the *esizei* and *meta set* commands. Extracted outcomes were organised a priori into five domains, clinical symptoms, functioning, quality of life and wellbeing, psychosocial outcomes, and physical health. For each eligible controlled study, standardised mean differences and corresponding 95% confidence intervals were calculated from reported means, standard deviations, and sample sizes. Effect sizes were coded so that positive values indicated greater improvement in the NBI group relative to the comparator condition. Where studies reported multiple measures within the same outcome domain, we selected the outcome most closely aligned with the domain definition and the study’s stated primary or most clinically relevant outcome. Where no primary outcome was specified, we prioritised validated and commonly used measures to maximise comparability across studies.

Where studies reported outcomes at multiple timepoints, the immediate post-intervention endpoint was prioritised for the main synthesis. Follow-up outcomes were extracted where available but were not pooled with immediate post-intervention effects. Where both endpoint and change scores were available, endpoint scores were prioritised for consistency across studies.

To avoid double-counting participants, each study contributed only one effect size to a given pooled analysis. Where studies reported multiple eligible intervention arms, comparator arms, outcome measures, or timepoints within the same analysis, a single effect size was selected according to the hierarchy described above. Multiple correlated effects from the same participants were not pooled within the same meta-analysis.

Random-effects models were applied to account for anticipated variability between studies. Heterogeneity was assessed using the I^2^ statistic and Cochran’s Q test; where substantial heterogeneity was identified (I^2^ > 75%), subgroup or sensitivity analyses were undertaken to explore potential sources of variation. Where meta-analysis was not feasible due to heterogeneity or data limitations, findings were synthesised narratively.

Formal assessment of publication bias was not conducted because fewer than 10 studies were included in the meta-analysis. Instead, potential publication bias was considered narratively, including whether small studies reported disproportionately large or significant effects and whether unpublished or grey literature was searched.

### 2.5. Qualitative Synthesis

Qualitative data were analysed through thematic synthesis (Thomas & Harden, 2008). This involved three stages: (1) line-by-line coding of the primary studies’ findings, (2) development and organisation of descriptive themes based on the codes, and (3) generation of analytical themes. Coding was conducted inductively, with codes actively identified and generated by the researcher through an iterative interaction with the data. All initial coding was completed by AG, and subsequent codes and themes were reviewed and refined in discussion with UZ and AON until consensus was reached. NVivo software (Lumivero, 2023, [version 15.2.0]) was used to support data management and organisation.

### 2.6. Integration

This review adopted a convergent segregated design (Hong et al., 2017), with quantitative and qualitative studies analysed separately using appropriate methods. Findings from both strands were then integrated to facilitate comparison and to explore how each type of evidence informs our understanding of the impact of NBIs. As the review was not explanatory in design, the qualitative findings were not used to interpret or explain the quantitative results but were instead reported alongside them to provide complementary insights.

### 2.7. Patient and Public Involvement

No patient or service-user contributors were involved in this review. Public and stakeholder input was provided by HF, a non-academic climate and sustainability contributor with experience in community mobilisation, clean air advocacy, green space access, and public climate engagement. Her input supported interpretation of the findings in relation to environmental justice, community participation, and the public relevance of nature-based approaches.

## 3. Results

### 3.1. Study Selection

The search identified 2060 articles; 456 were excluded after deduplication, and 97 were excluded during the full-text screening. Of the included 20 articles, a further eight were identified from backwards and forwards citation searching, resulting in a final sample of 28 studies (Supplementary Materials p. 25; Figure S1). Quantitative findings are presented first, structured by the five predefined outcome domains (clinical symptoms, functioning, quality of life and wellbeing, psychosocial outcomes, and physical health). These are followed by the qualitative synthesis of themes, after which both strands are considered together in a narrative comparison.

### 3.2. Study Characteristics

The 28 included studies comprised 14 quantitative (Sisman et al., 2020; Pedersen et al., 2011, 2012b; Keenan et al., 2021; Barton et al., 2012; Atta et al., 2025; Zhu et al., 2016; Walter et al., 2023; Wahrborg et al., 2014; Shimizu et al., 2023; Oh et al., 2018; Müller et al., 2025; He et al., 2020; Berget et al., 2007), 10 qualitative (Wästberg et al., 2021; Pedersen et al., 2012a; Pálsdóttir et al., 2021; Leighton et al., 2021; Iancu et al., 2014; Fieldhouse, 2003; Cooley et al., 2021; Cerwén et al., 2016; Barley et al., 2012; Joung et al., 2025), and four mixed methods studies (Kam & Siu, 2010; Iwata et al., 2016; Vujcic Trkulja et al., 2021; Siu et al., 2020), published between 2003 and 2025. Sample sizes ranged from 6 to 781 participants; and interventions were delivered in both inpatient (He et al., 2020; Zhu et al., 2016; Leighton et al., 2021; Pálsdóttir et al., 2021; Joung et al., 2025; Atta et al., 2025; Iwata et al., 2016) and outpatient/community rehabilitation settings (Oh et al., 2018; Pedersen et al., 2011, 2012a, 2012b; Shimizu et al., 2023; Walter et al., 2023; Wahrborg et al., 2014; Cooley et al., 2021; Siu et al., 2020; Fieldhouse, 2003; Vujcic Trkulja et al., 2021; Iancu et al., 2014; Wästberg et al., 2021; Cerwén et al., 2016; Barley et al., 2012; Müller et al., 2025; Kam & Siu, 2010; Sisman et al., 2020; Keenan et al., 2021; Barton et al., 2012) with one spanning both (Berget et al., 2008). Interventions were mostly horticulture-based (50.0%) (He et al., 2020; Oh et al., 2018; Zhu et al., 2016; Wahrborg et al., 2014; Siu et al., 2020; Fieldhouse, 2003; Pálsdóttir et al., 2021; Vujcic Trkulja et al., 2021; Wästberg et al., 2021; Cerwén et al., 2016; Barley et al., 2012; Kam & Siu, 2010; Atta et al., 2025; Sisman et al., 2020) followed by care farming (25.0%) (Pedersen et al., 2011, 2012a, 2012b; Shimizu et al., 2023; Berget et al., 2008; Iancu et al., 2014; Joung et al., 2025); walking or exercise-focused (17.9%) (Walter et al., 2023; Cooley et al., 2021; Keenan et al., 2021; Iwata et al., 2016; Barton et al., 2012); and integrated multimodal programmes combining horticulture; arts; or mindfulness practices (7.1%) (Müller et al., 2025; Leighton et al., 2021). Duration ranged from a single 6 h session to 23 weeks; with frequency from once weekly to six sessions per week. Most studies had a control group (53.6%) (He et al., 2020; Oh et al., 2018; Shimizu et al., 2023; Walter et al., 2023; Wahrborg et al., 2014; Berget et al., 2008; Pedersen et al., 2012a; Vujcic Trkulja et al., 2021; Müller et al., 2025; Siu et al., 2020; Iancu et al., 2014; Keenan et al., 2021; Kam & Siu, 2010; Atta et al., 2025; Barton et al., 2012; Sisman et al., 2020) that either received treatment-as-usual (28.6%) (Atta et al., 2025; Sisman et al., 2020; Pedersen et al., 2012b; He et al., 2020; Siu et al., 2020; Wahrborg et al., 2014; Müller et al., 2025; Berget et al., 2008) or non-nature-based group activities (25.0%) (Oh et al., 2018; Shimizu et al., 2023; Vujcic Trkulja et al., 2021; Iancu et al., 2014; Keenan et al., 2021; Kam & Siu, 2010; Barton et al., 2012).

Participants had a broad range of psychiatric diagnoses, most frequently depression (56.7%) (Pedersen et al., 2011, 2012a, 2012b; Walter et al., 2023; Wahrborg et al., 2014; Cooley et al., 2021; Fieldhouse, 2003; Pálsdóttir et al., 2021; Vujcic Trkulja et al., 2021; Iancu et al., 2014; Wästberg et al., 2021; Cerwén et al., 2016; Barley et al., 2012; Müller et al., 2025; Kam & Siu, 2010; Keenan et al., 2021; Iwata et al., 2016) and schizophrenia (43.3%) (He et al., 2020; Oh et al., 2018; Zhu et al., 2016; Shimizu et al., 2023; Berget et al., 2008; Cooley et al., 2021; Siu et al., 2020; Fieldhouse, 2003; Iancu et al., 2014; Kam & Siu, 2010; Joung et al., 2025; Atta et al., 2025; Sisman et al., 2020). Mean ages spanned the 20s to 60s, and both men and women were included. Ethnicity was rarely reported (10.7%) (Walter et al., 2023; Cooley et al., 2021; Fieldhouse, 2003).

Detailed study characteristics are provided in Table 1, and detailed intervention and comparator characteristics (content, duration, frequency, assessment windows) are reported in the Supplementary Materials (File S2 pp. 24–29; Table S1). 

### 3.3. Quality Appraisal

CASP appraisal indicated that four of five randomised controlled trials were of low quality and one was of moderate quality, while cohort studies ranged from low (n = 2) to moderate (n = 3) and high quality (n = 1). In contrast, all ten qualitative studies met at least 80% of applicable CASP criteria and were therefore rated as high quality.

The most common limitation across quantitative studies was inadequate blinding. Although participant and investigator blinding is inherently difficult in NBI research, outcome assessor blinding was inconsistently reported or implemented. Most RCTs also did not fully account for all participants at study completion, and several reported baseline group differences, limiting confidence in between-group comparisons. Cohort studies were similarly limited by short or inadequate follow-up and incomplete adjustment for potential confounding variables. Qualitative studies were generally stronger methodologically, although most did not adequately reflect on the researcher–participant relationship.

Detailed quality appraisal is provided in the Supplementary Materials (File S4 pp. 41–45; Quality appraisal).

### 3.4. Results of Individual Studies (Non-Pooled Domains)

#### 3.4.1. Clinical Symptoms

Sixteen studies examined clinical symptom outcomes, encompassing horticulture (He et al., 2020; Oh et al., 2018; Zhu et al., 2016; Wahrborg et al., 2014; Vujcic Trkulja et al., 2021; Siu et al., 2020; Kam & Siu, 2010), care farming (Pedersen et al., 2011, 2012b; Berget et al., 2008; Shimizu et al., 2023), outdoor-activity-based interventions (Walter et al., 2023; Keenan et al., 2021; Iwata et al., 2016; Barton et al., 2012) and integrating alternative therapies such as mindfulness (Müller et al., 2025).

Four out of seven studies in participants diagnosed with schizophrenia [three quantitative studies (Zhu et al., 2016; Oh et al., 2018; He et al., 2020) and one mixed method study (Kam & Siu, 2010)] reported improvements in clinical symptoms following structured horticultural programmes (Zhu et al., 2016; Oh et al., 2018; He et al., 2020; Kam & Siu, 2010). Among individuals with depression or stress-related disorders, Währborg and colleagues observed that horticultural participation significantly reduced healthcare consumption, reflected in fewer outpatient visits and inpatient psychiatric admissions, although sick-leave status remained unchanged (Wahrborg et al., 2014). In contrast, Siu and colleagues found no significant reductions in stress or anxiety among participants with depression, suggesting possible heterogeneity in programme design or intensity (Siu et al., 2020).

Outdoor activity interventions also yielded benefits (Keenan et al., 2021; Barton et al., 2012; Walter et al., 2023; Iwata et al., 2016). In participants with depression, both surf and hike therapies were effective adjunctive interventions for reducing depressive symptoms (Walter et al., 2023). Mindfulness and relaxation-based nature interventions were associated with significant improvements in depressed mood that were maintained at three-month follow-up, suggesting sustained effects of guided contemplative practice in natural settings (Müller et al., 2025). Animal-assisted interventions showed more variable outcomes. Pedersen and colleagues found that 12-week farm animal-assisted interventions decreased anxiety and depression levels in participants with depression (Pedersen et al., 2011, 2012b), whereas a one-day sheep-rearing activity did not improve anxiety among individuals with schizophrenia (Shimizu et al., 2023). Berget and colleagues reported that greater engagement in a 12-week farm work intervention correlated with anxiety reduction in affective disorder participants, though this effect was not generalisable to the full sample (Berget et al., 2007).

Taken together, horticultural and structured outdoor activity interventions show the most consistent benefits for clinical symptoms. Findings across studies of animal-assisted and brief experiential programmes are mixed, possibly reflecting differences in intervention duration, population, and methodological rigour. Detailed pre-post means, SDs, and *p* values for all clinical outcomes are presented in Table 2.

#### 3.4.2. Quality of Life and Wellbeing

Seven out of twenty-eight studies assessed quality of life and wellbeing outcomes, including horticulture-based [two quantitative studies (He et al., 2020; Atta et al., 2025) and two mixed method studies (Kam & Siu, 2010; Siu et al., 2020)], animal-assisted (one quantitative study, Berget et al., 2008), exercise-focused (one quantitative study, Keenan et al., 2021), and mindfulness-based interventions (one quantitative study, Müller et al., 2025).

Horticultural programmes yielded mixed results. Some studies reported significant improvements in mental wellbeing following participation in a structured horticultural programme among adults with depression (Siu et al., 2020) and psychotic disorders (Atta et al., 2025) whereas others found no significant change in wellbeing outcomes among participants with schizophrenia (Siu et al., 2020) and other mental disorders (Kam & Siu, 2010).

Mindfulness-based nature interventions were associated with selective benefits. Müller and colleagues (Müller et al., 2025) observed increased self-compassion following a nature-based mindfulness and relaxation programme for individuals with depression, though no corresponding improvement in mindfulness scores was detected. In contrast to nature walks, an animal-assisted intervention involving farm activities did not produce significant changes in wellbeing among participants with psychiatric disorders (Berget et al., 2008).

Overall, findings indicate that while nature-based interventions can enhance aspects of wellbeing such as self-compassion and perceived mental wellbeing, effects are inconsistent across populations and intervention types. Study-level quality of life and wellbeing outcomes are summarised in the Supplementary Materials (File S3 pp. 30–33; Table S3).

#### 3.4.3. Psychosocial

Three out of four quantitative studies investigating farm animal-assisted interventions reported an increase in self-efficacy (Berget et al., 2008; Pedersen et al., 2011, 2012b). Berget and colleagues found a significant improvement in coping abilities that was maintained at 6 months. Moreover, Barton and colleagues found an improvement in self-esteem in participants attending a green exercise group (Barton et al., 2012). Siu and colleagues examined social exchange among participants; however, no significant differences were observed (Siu et al., 2020). Study-level psychosocial outcomes are summarised in the Supplementary Materials (File S3 pp. 33–36; Table S4).

#### 3.4.4. Functional Outcomes

Four out of five studies [three quantitative studies (He et al., 2020; Atta et al., 2025; Sisman et al., 2020) and one mixed method study (Siu et al., 2020)] found that horticulture enhanced the social functioning of participants with schizophrenia (He et al., 2020; Siu et al., 2020; Atta et al., 2025; Sisman et al., 2020). Furthermore, Siu and colleagues reported that horticulture significantly promoted participants’ engagement in meaningful activities, although no significant changes in affect were found (Siu et al., 2020). Study-level functional outcomes are summarised in the Supplementary Materials (File S3 pp. 36–37 Table S2).

#### 3.4.5. Physical Health

Finally, one study reported the physical health benefits of NBIs (Shimizu et al., 2023). Shimizu and colleagues found that a one-day sheep-rearing experiential programme significantly increased salivary testosterone levels without elevating cortisol or anxiety, suggesting potential benefits for motivation and stress regulation in individuals with schizophrenia. Physical health outcomes are summarised in the Supplementary Materials (File S3 p. 38; Table S5).

### 3.5. Results of Syntheses

**Figure 1:**
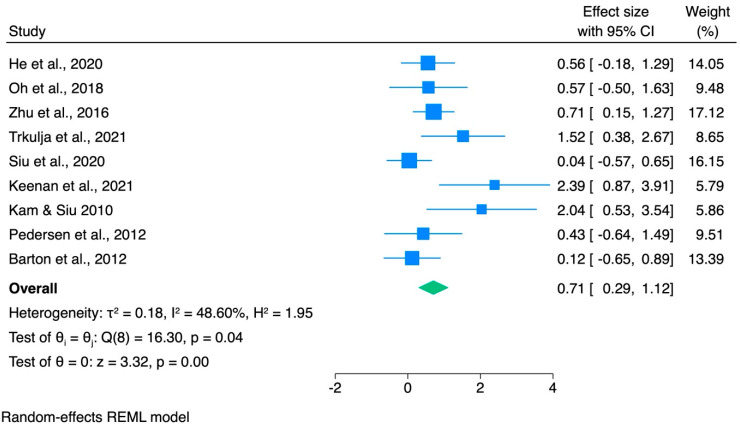
Random-effects meta-analysis of nature-based interventions versus control on mental-health and functional outcomes. Each square represents an individual study’s (He et al., 2020; Oh et al., 2018; Zhu et al., 2016; Vujcic Trkulja et al., 2021; Siu et al., 2020; Keenan et al., 2021; Kam & Siu, 2010; Pedersen et al., 2012b; Barton et al., 2012) standardised mean difference (Hedges’ g) with 95% confidence intervals. Square size reflects study weight, and the diamond represents the pooled random-effects estimate (SMD = 0·71 [95% CI 0·29–1·12]; *p* = 0·001). Between-study heterogeneity was moderate (τ^2^ = 0·18; I^2^ = 48·6%; Q(8) = 16·30; *p* = 0·04).

Across nine studies (He et al., 2020; Oh et al., 2018; Zhu et al., 2016; Vujcic Trkulja et al., 2021; Siu et al., 2020; Keenan et al., 2021; Kam & Siu, 2010; Pedersen et al., 2012b; Barton et al., 2012), a random-effects meta-analysis showed that the intervention was associated with improvements in clinical symptoms than control conditions (pooled effect size 0.71 [95% CI 0.29–1.12]; *p* = 0.0009; see Figure 1). Study-specific effects ranged from 0.04 to 2.39, and between-study heterogeneity was moderate (τ^2^ = 0.18; I^2^ = 48.6%; Q(8) = 16.30; *p* = 0.038). All included studies were controlled trials, comprising both randomised and non-randomised designs. In an exploratory random-effects meta-regression including NBI type and trial design, neither exercise-focused (β = 0.76 [95% CI −1.63 to 3.14]; *p* = 0.54) nor horticultural programmes (β = 0.46 [95% CI −1.53 to 2.45]; *p* = 0.65) differed significantly from care farming, and randomised controlled trials did not differ significantly from non-randomised controlled studies (β = 0.31 [95% CI −1.03 to 1.66]; *p* = 0.65). As a set, these moderators did not explain between-study variation (Wald χ^2^(3) = 0.45; *p* = 0.93; R^2^ = 0%), and substantial residual heterogeneity remained (residual τ^2^ = 0.56; I^2^ = 75.7%; Q_res(5) = 16.16; *p* = 0.006). However, this meta-regression included only nine studies and three moderators and was therefore underpowered; the findings should be interpreted as exploratory and hypothesis-generating rather than confirmatory.

Finally, a sensitivity analysis excluding the two studies with the largest effect sizes (Keenan et al., 2021; Kam & Siu, 2010) attenuated the pooled effect, but the association remained statistically significant (SMD 0.48 [95% CI 0.17–0.79]; *p* = 0.0025). Heterogeneity was substantially reduced (τ^2^ = 0.018; I^2^ = 10.0%; Q(6) = 6.74; *p* = 0.345), suggesting that these two studies contributed meaningfully to between-study heterogeneity, but did not fully account for the overall direction of effect.

### 3.6. Qualitative Findings

**Figure 2:**
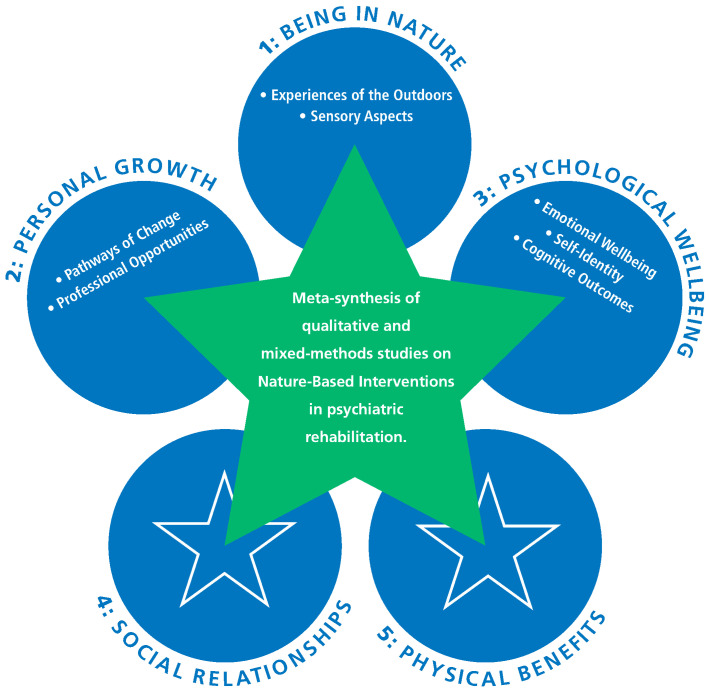
Thematic map of five key themes identified. The central concept is surrounded by five overarching themes, Being in Nature, Personal Growth, Psychological Wellbeing, Social Relationships and Physical Benefits, with associated subthemes, illustrating how nature-based interventions may support recovery processes.

Of the included studies ten were qualitative (Barley et al., 2012; Cerwén et al., 2016; Cooley et al., 2021; Fieldhouse, 2003; Iancu et al., 2014; Joung et al., 2025; Leighton et al., 2021; Pálsdóttir et al., 2021; Pedersen et al., 2012a; Wästberg et al., 2021), and four employed a mixed methods design (Iwata et al., 2016; Kam & Siu, 2010; Siu et al., 2020; Vujcic Trkulja et al., 2021). A meta-synthesis of their findings yielded five overarching themes and are shown on a thematic map (See Figure 2). Two themes, “Social Relationships” and “Physical Benefits”, did not yield subthemes. The data coded to “Social Relationships” were consistent enough that further splitting was not needed, while fewer studies discussed physical benefits, limiting the development of subthemes. Exemplar quotes underpinning themes and subthemes are provided in the Supplementary Materials (File S4 pp. 38–39; Table S6). Quotes are referenced in the main text using the source codes S1–S26 (e.g., “S1”).

The theme “Psychological Wellbeing” was present across all included studies. Participants consistently reported improvements in mood (S1, S2, S14), reductions in stress and anxiety (S15), enhanced emotional expression, and greater confidence in coping (S13). NBIs supported a stronger sense of self (S17), reinforcing self-efficacy and self-worth (S16), and in some cases fostering existential reflections on connectedness (S18) and purpose. Cognitive outcomes (S19), including improvements in attention (S20), memory, temporal orientation, and clarity of thought (S21), were also highlighted, often linked to sensory engagement with the environment. These accounts illustrate how NBIs address core psychiatric symptoms while also enhancing coping and resilience.

The theme “Being in Nature” was reported consistently across nine studies. Participants described the value of fresh air, open space (S1, S2), and closeness to plants (S3, S4, S5) and animals (S5), often linking these experiences to positive emotions and motivation (S12). Tangible outcomes of horticulture, such as fresh produce or flowers, were associated with responsibility and pride. Negative experiences, including allergies, tiredness, or low mood linked to weather, were less common but highlighted variability in response. Sensory dimensions were central (S6, S18), natural scents (S4, S6) and sounds (S5) were perceived as calming, restorative, and evocative of positive memories, while traffic noise or other intrusions were disruptive (S2). These findings suggest that both material and sensory features of nature underpin the therapeutic potential of NBIs, particularly in supporting attention and emotional regulation.

The theme “Personal Growth”, identified in nine studies, reflected processes of individual change (S7) and recovery (S12). Participation enabled acceptance of illness (S8), interruption of restrictive routines, and opportunities to experience aspects of “ordinary life.” Lifestyle changes such as reduced medication or substance use (S9), and the adoption of new hobbies (S10, S11) were reported. Participants also developed a sense of environmental ownership, taking pride in their contributions. NBIs were further described as offering transferable skills and, in some cases, pathways to professional opportunities (S10, S11, S12). These findings align with core recovery principles, including autonomy, identity, and hope.

The theme “Social Relationships” appeared in ten studies and reflected the relational opportunities created through NBIs (S22, S23). Participants described forming new friendships, cooperating with others, and experiencing empathy and belonging (S24). Some connections extended beyond the intervention, reinforcing social support networks and providing new roles. At the same time, participants valued the balance NBIs offered between social participation and solitude in nature. These findings highlight the dual role of NBIs in fostering social functioning while allowing personal reflection.

Finally, the theme “Physical Benefits”, reported in two studies, highlighted perceived gains in strength, energy (S25), and vitality. For some participants, the physicality of outdoor activities was described as liberating (S26), contributing to a broader sense of wellbeing.

Across studies, participants consistently characterised NBIs as interventions that operate across psychological, social, sensory, and physical domains, engaging multiple pathways relevant to psychiatric rehabilitation. These findings should be interpreted in the context of diverse clinical and cultural settings, and with consideration of variations in reporting styles and researcher reflexivity.

### 3.7. Integration

When considered together, the quantitative and qualitative strands showed both broad convergences, and some divergence across intervention types and outcome domains. Consistent with our convergent segregated approach, we compared findings across strands narratively rather than through a formal convergence matrix.

Improvements in clinical symptoms identified in the narrative synthesis were consistently reflected in qualitative accounts of enhanced psychological wellbeing, further supporting the “Psychological Wellbeing” and “Personal Growth” themes. Studies which showed quantitative reductions in depression (Müller et al., 2025; Pedersen et al., 2011, 2012b; Walter et al., 2023), anxiety (Berget et al., 2007), and psychiatric symptom severity (Zhu et al., 2016; Oh et al., 2018; He et al., 2020; Kam & Siu, 2010) observed particularly in horticultural and structured outdoor activity interventions often involved the same interventions in which participants described feeling calmer (Cooley et al., 2021; Siu et al., 2020), coping better (Iancu et al., 2014; Leighton et al., 2021), and being more active in everyday life (Barley et al., 2012; Cooley et al., 2021; Siu et al., 2020). Similarly, quantitative improvements in social and functional outcomes, including self-efficacy (Berget et al., 2008), coping ability (Berget et al., 2008), and social functioning (He et al., 2020; Siu et al., 2020), aligned with qualitative themes of “Personal Growth” and “Social Relationships,” where participants reported greater confidence (Fieldhouse, 2003; Wästberg et al., 2021), renewed identity (Barley et al., 2012; Iancu et al., 2014), meaningful social participation (Iancu et al., 2014; Kam & Siu, 2010), and engagement in ordinary life activities (Cooley et al., 2021; Pedersen et al., 2012a).

The qualitative findings also helped contextualise why certain intervention types may have produced stronger quantitative effects. Themes related to emotional restoration, sensory engagement, responsibility, and connectedness to nature were particularly prominent in horticultural and outdoor activity programmes, which were also associated with the most consistent quantitative improvements.

At the same time, some divergence between strands was observed. “Being in Nature” emerged as a consistent theme across qualitative studies, yet this dimension was absent from quantitative measurement frameworks. No included quantitative study systematically assessed sensory or material features of the natural environment as potential mechanisms of change. In areas where quantitative findings were mixed or limited, such as quality of life and some animal-assisted or brief programmes, qualitative accounts still described changes in mood (Iancu et al., 2014; Pedersen et al., 2012a), motivation (Iancu et al., 2014), and connection with others (Iancu et al., 2014; Joung et al., 2025; Pedersen et al., 2012a). These discrepancies may reflect limitations in the sensitivity of standardised outcome measures to capture subjective or recovery-oriented changes that participants considered important. Looking across both types of evidence suggests some overlap between what was measured and what participants reported and also points to areas where people describe benefits that have not been clearly shown in quantitative results yet.

Overall, integrating both strands suggests that NBIs may influence psychiatric rehabilitation across multiple interconnected domains, including symptom reduction, emotional regulation, social connectedness, identity, and functional recovery.

## 4. Discussion and Conclusions

This mixed methods review synthesised quantitative and qualitative evidence on NBIs for people with psychiatric disorders, filling a critical gap in the recent literature (Jessen et al., 2025; Brandt et al., 2026) by integrating clinical efficacy with functional and experiential outcomes. Quantitative findings showed the most consistent benefits for structured horticulture and guided outdoor activities (symptoms, mood, functioning, and in some cases healthcare use), with more mixed results for animal-assisted and brief experiential programmes. However, qualitative evidence opened the black box of these effects, pointing to transformations in sense of self and social connection that go beyond simple symptom reduction.

Across outcome domains, quantitative studies suggested that some NBIs were associated with improvements in clinical symptoms, functioning, and selected psychosocial outcomes, although effects on quality of life and wellbeing were more variable and not always tested against appropriate control conditions. Evidence was most consistent for more structured and sustained programmes, particularly horticultural and guided outdoor activity interventions, which were also the most frequently studied approaches. Overall, the pooled estimate from controlled studies favoured NBIs over comparator conditions, and exploratory analyses did not indicate clear differences in effect size by trial design. A sensitivity analysis excluding the two studies with the largest effect sizes attenuated the pooled estimate from 0.71 to 0.48, although the association remained statistically significant (SMD 0.48 [95% CI 0.17–0.79]; *p* = 0.0025). Heterogeneity was substantially reduced (I^2^ = 10.0%; τ^2^ = 0.018), suggesting that these two studies contributed meaningfully to between-study variability. However, confidence in the meta-analytic estimate is limited by study quality, as four of the five RCTs included in the quantitative synthesis were rated as low quality using CASP. The pooled effect should therefore be interpreted as promising but cautious, rather than definitive improvements in clinical symptoms identified in the quantitative synthesis were strongly reflected in qualitative accounts of enhanced psychological wellbeing. Similarly, quantitative improvements in social and functional outcomes, including self-efficacy (Berget et al., 2008; Pedersen et al., 2011), coping ability (Berget et al., 2008), and social functioning (He et al., 2020; Siu et al., 2020) aligned with qualitative results: participants reported enhancements in self-confidence (Fieldhouse, 2003; Wästberg et al., 2021), a renewed identity (Barley et al., 2012; Iancu et al., 2014), social participation (Iancu et al., 2014; Kam & Siu, 2010), and engagement in ordinary and meaningful activities (Cooley et al., 2021; Pedersen et al., 2012a).

At the same time, some divergence between quantitative and qualitative findings emerged. In domains where quantitative evidence was inconsistent or limited, particularly regarding quality of life outcomes and animal-assisted interventions, participants nevertheless described meaningful improvements in mood, motivation, and social connectedness.

Taken together with qualitative accounts, the findings suggest that NBIs may contribute to recovery via multi-domain pathways that are not consistently captured by symptom-focused measures. Indeed, the qualitative evidence complemented the quantitative findings by showing the lived experiences and potential mechanisms underlying observed clinical improvements, while also identifying benefits that remain insufficiently captured within existing quantitative measures. Heterogeneity likely reflects differences in context and delivery, underscoring the need for better-specified interventions and harmonised, recovery-relevant outcomes.

Collectively, this evidence suggests that NBIs may function as recovery-oriented approaches that can be integrated alongside existing psychiatric treatments. Consistent qualitative reports of reduced stress, emotional relief, “clearer heads”, improved sleep, and renewed motivation align with theoretical models emphasising stress reduction (Bratman et al., 2012; Ulrich, 1984), attention restoration (Bratman et al., 2012), and behavioural activation in natural environments. However, the potential mechanisms are likely broader than these classic psychological frameworks. Outdoor NBIs may support circadian entrainment through daylight exposure, routine, and daytime activity, which is relevant given the role of circadian rhythm disruption in depression and other psychiatric disorders (Monteleone et al., 2011). They may also influence HPA-axis and stress-regulatory systems, given evidence of altered cortisol dynamics in chronic psychiatric disorders, including schizophrenia (Aas et al., 2019). More speculatively, improvements in sleep and reductions in stress—both repeatedly described in the qualitative synthesis—may engage neurobiological pathways, including glymphatic clearance, that are increasingly recognised as relevant to psychiatric disorders (Barlattani et al., 2025). These converging psychological and biological pathways position NBIs not simply as adjunctive wellbeing activities, but as potentially biologically informed rehabilitation approaches that may affect symptoms, functioning, identity, social participation, and physiological regulation.

Furthermore, participants’ accounts of reconnecting with an “inner self”, taking on responsibilities, and developing new skills or vocational aspirations resonate strongly with recovery-oriented care, where autonomy, purpose, and social inclusion are central outcomes (Leamy et al., 2011; Anthony, 1993). Importantly, some participants described benefits even when symptom change was modest, suggesting that conventional clinical scales may under capture valued gains such as confidence, hope, and a sense of belonging (Andresen et al., 2003).

There are, however, several limitations. Despite our meta-analysis of horticultural interventions, the number of controlled studies was small, and the pooled sample size was limited, reducing precision and restricting the scope for subgroup, sensitivity, and publication-bias analyses. We were therefore unable to formally assess publication bias, meaning that small-study effects and selective publication cannot be excluded. The quality of RCT evidence was also variable, with several studies affected by small samples, short follow-up, incomplete reporting, limited blinding, and potential risks of bias. Diagnostic heterogeneity was substantial, with studies including participants with different psychiatric diagnoses, illness stages, symptom profiles, and levels of functional impairment. This limits conclusions about which psychiatric populations are most likely to benefit from specific NBI formats. There was also considerable variation in how NBIs were operationalised, including differences in intervention content, dose, duration, setting, facilitation, group structure, and comparator conditions. As a result, direct comparisons between intervention types should be interpreted cautiously. We also excluded virtual or simulated nature, which may be relevant for people with limited access to outdoor spaces, and there was considerable variation in how NBIs were operationalised. Moreover, a potential limitation of this review is the use of CASP tools for quality appraisal across all included study designs. While this enabled a consistent and integrated approach across heterogeneous methodologies, CASP provides a broader critical appraisal framework rather than a domain-based risk-of-bias assessment specific to randomised controlled trials, such as the Cochrane RoB-2 tool. No patient or service-user contributors with lived experience of psychiatric disorders were involved, which is a limitation given the qualitative synthesis’ focus on lived experience.

Several implications for future research and practice emerge from the breadth of diagnoses, intervention types, and intervention intensities included in this review. The most consistent evidence was found for structured and sustained horticultural programmes, particularly among people with schizophrenia-spectrum disorders, where several studies reported improvements in clinical symptoms and social functioning. There was also some evidence that longer farm-based or animal-assisted interventions may benefit people with depression or affective disorders, particularly when delivered over several weeks, whereas very brief or single-session activities appeared less likely to produce measurable change. Evidence for green exercise, mindfulness-based nature activities, and other outdoor experiential programmes was more limited and heterogeneous, making it difficult to draw firm disorder-specific conclusions.

These patterns suggest that intervention structure and intensity may be important. Programmes delivered over multiple sessions, with clear therapeutic or rehabilitative components, appeared more likely to show benefits than brief, low-intensity, or one-off activities. However, the current evidence base remains too small and heterogeneous to determine the optimal dose, duration, or modality of NBIs for specific psychiatric populations. Future studies should therefore compare different NBI formats and intensities, and examine whether mechanisms such as social contact, responsibility, sensory engagement, physical activity, routine, and connection with nature differentially benefit particular diagnostic groups. Priorities include adequately powered, high-quality RCTs with standardised recovery-relevant outcomes and longer follow-up, alongside mixed methods designs that clarify mechanisms, implementation processes, and lived experience.

With respect to implementation, NBIs may be particularly well suited to community mental health and psychiatric rehabilitation settings, where structured, sustained programmes can complement existing care pathways. For people with schizophrenia and other severe mental illnesses, participation in nature-based activities may offer value beyond symptom reduction. As discussed above, NBIs can provide opportunities to occupy non-patient roles (Iancu et al., 2014; Pedersen et al., 2012a), “to break down the confinement of the ‘four walls’” (Leighton et al., 2021), engage with the social world, and rebuild a sense of identity and agency that institutional settings can constrain. This aligns with the CHIME framework (Leamy et al., 2011) and warrants dedicated investigation. Encouragingly, integration of NBIs into NHS mental health services is already underway: South London and Maudsley NHS Foundation Trust, for instance, has recently introduced nature walks, community nature volunteering, and ward garden programmes across several services (South London and Maudsley NHS Foundation Trust, 2025), suggesting that implementation is feasible and increasingly prioritised. Overall, NBIs show promise as recovery-oriented, low-intensity interventions within mental healthcare, particularly when delivered in structured and sustained formats. Our findings point to the potential utility of integrating NBIs within flexible, person-centred psychiatric rehabilitation pathways, especially where access to safe natural spaces can be ensured and interventions can be aligned with individual preferences and goals. However, given the heterogeneity and methodological limitations of the current evidence, further high-quality research is needed to clarify which intervention approaches are most effective for specific psychiatric populations and contexts.

## Figures and Tables

**Table 1:** Sample characteristics of examined studies. NBI = nature-based intervention; NR = not reported; RCT = randomised controlled trial; ICD-10 = International Classification of Diseases, 10th Revision; DSM-IV = Diagnostic and Statistical Manual of Mental Disorders, 4th Edition; MINI = Mini-International Neuropsychiatric Interview; BDI-IA = Beck Depression Inventory, version IA; PTSD = post-traumatic stress disorder.

Author(s), Year	Country	Design, Analysis	NBI	Diagnosis (Criteria)	Duration	Frequency	N	Gender (M/F/O)	Age M (SD)	Ethnicity
**Atta et al. (2025)**	Egypt	Quantitative; Randomised Controlled Trial	Horticulture	Schizophrenia; bipolar disorder (Clinical diagnosis from records)	8 weeks (approximately 56 days).	1×/week × 30 min	120	NR	NR	NR
**He et al. (2020)**	China	Quantitative; Randomised Controlled Trial	Horticulture	Schizophrenia (ICD-10)	6 weeks	1×/week × 60 min	60	F	NR	NR
**Sisman et al. (2020)**	Turkey	Quantitative; Randomised Controlled Trial	Horticulture	Schizophrenia (Clinical diagnosis from records)	6 weeks total (programme conducted over six sessions spanning 6 weeks)	1×/week × NR (mix of 50–90 min across sessions)	80	48/32	41.20 (9.19)	NR
**Oh et al. (2018)**	South Korea	Quantitative; Non-Randomised Controlled Trial	Horticulture	Schizophrenia (NR)	12 weeks	1×/week × ~120 min	28	20/8	NR	NR
**Zhu et al. (2016)**	China	Quantitative; Randomised Controlled Trial	Horticulture	Schizophrenia (ICD-10)	12 weeks	3×/week × 90 min	110	48/52	46.5 (9.0)	NR
**Wahrborg et al. (2014)**	Sweden	Quantitative; Retrospective Cohort Study	Horticulture	Exhaustion syndrome; depression; PTSD 9 ICD-10)	12 weeks	NR (individually adapted)	781	89/692	46.25 (9.69)	NR
**Pedersen et al. (2012b)**	Norway	Quantitative; Randomised Controlled Trial	Care farming	Major depressive disorder (BDI-IA ≥ 14; DSM-IV major depression—MINI in subsample)	12 weeks (≈84 days)	2 × per week, 1.5–3 h	29	6/23	37.8 (NR)	NR
**Pedersen et al. (2011)**	Norway	Quantitative; Within-Subject, Exploratory, Observational Intervention Study	Care farming	Depression (DSM-IV and BDI-IA)	12 weeks	2×/week × 1.5–3 h	14	3/11	37.4 (NR)	NR
**Berget et al. (2008)**	Norway	Quantitative; Randomised Controlled Trial	Care farming	Schizophrenia/schizotypal; affective; anxiety/stress-related; personality disorders (ICD-10)	12 weeks	2×/week × 3 h	90	31/59	34.7 (10.7)	NR
**Shimizu et al. (2023)**	Japan	Quantitative; Non-Randomised Controlled Trial	Care farming	Schizophrenia (DSM-IV-TR)	1 day	1× (day) × 10:30–15:00	14	9/5	56 (13.79)	NR
**Walter et al. (2023)**	USA	Quantitative; Randomised Controlled Trial	Exercise Focused	Major depressive disorder (MINI-7)	6 weeks	1×/week × 3–4 h	96	46/50	28.1 (5.6)	White 40 (41.7%); Multiracial 19 (19.8%); Hispanic, Latinx, or Spanish origin 18 (18.8%); Black or African American 15 (15.6%); Asian or Asian American/Native American or Alaska Native 4 (4.2%)
**Keenan et al. (2021)**	Ireland	Quantitative; Randomised Controlled Trial	Exercise Focused	Anxiety; depression (NR)	5 consecutive days	5 days × 30 min	50	20/30	40.34 (12.65)	NR
**Barton et al. (2012)**	UK	Quantitative; Non-Randomised Controlled Trial	Exercise Focused	Mixed mental health diagnoses (mood, anxiety, psychotic, substance-related) (Clinician-assigned DSM-IV Axis I diagnoses)	6 weeks; one 45 min walk per week (≈6 sessions total).	1×/week × 45 min	53	20/33	53.0 (15.4)	NR
**Müller et al. (2025)**	Germany	Quantitative; Non-Randomised Controlled Trial	Integrating Alternative Therapies	Depression (NR)	Once a week	1×/week × 4 h (Centre 1); NR (Centre 2)	227	87/134/1	51.66 (NR)	NR
**Vujcic Trkulja et al. (2021)**	Serbia	Mixed Methods; Non-Randomised Controlled Trial, and On-Site Ethnographic Observations	Horticulture	Adjustment disorder, reaction to severe stress, anxiety, depression (ICD-10)	5 weeks	3×/week × 1 h	27	19/8	43.92 (10.16)	NR
**Siu et al. (2020)**	China	Mixed Methods; Randomised Controlled Trial	Horticulture	Schizophrenia; Other Psychiatric Illness (NR)	8 weeks	1×/week × 50 min	82	37/45	50.3 (9.6)	NR
**Kam and Siu (2010)**	Hong Kong	Mixed Methods; Single-blind RCT with embedded qualitative evaluation	Horticulture	Schizophrenia spectrum; bipolar disorder; major depression (NR)	2 weeks (10 consecutive days)	1×/day × 1 h	24	17/7	44.3 (11.6)	NR
**Iwata et al. (2016)**	Ireland	Mixed Methods; Within-Subject, Observational Intervention Study with Thematic Analysis	Exercise Focused	Depression; bipolar disorder; anxiety disorder; other (NR)	13 weeks	1×/week × 2 h	15	3/12	47.0 (NR)	NR
**Pálsdóttir et al. (2021)**	Sweden	Qualitative; Interpretative phenomenological analysis	Horticulture	Stress-related mental disorders, i.e., exhaustion disorder or depression (ICD-10)	12 weeks	4×/week × 4 h	59	9/50	45.5 (NR)	NR
**Wästberg et al. (2021)**	Sweden	Qualitative; Narrative methodology	Horticulture	Depression; Anxiety; stress-related disorders (NR)	NR	2×/week × ~3 h	8	1/7	NR	NR
**Cerwén et al. (2016)**	Sweden	Qualitative; Interpretative phenomenological analysis	Horticulture	Adjustment and stress-related disorders (including exhaustion disorder and depression) (ICD-10)	12 weeks	4×/week × 4 h	59	9/50	NR	NR
**Barley et al. (2012)**	UK	Qualitative; Constant Comparison and Thematic Analysis	Horticulture	Depression; bipolar disorder; mixed anxiety and depression, multiple sclerosis; psychotic disorder; social isolation (NR)	Interviewees were broadly representative of project users. Attendance ranged from 6 weeks to 4 years (n = 12).	6 garden + 2 arts/week × 2.5–3 h	16	9/7	NR	NR
**Fieldhouse (2003)**	UK	Qualitative; Not reported	Horticulture	Mixed diagnoses (psychotic, mood, anxiety, substance misuse, undetermined) (NR)	(~14 years combined).	1×/week × NR	9	6/3	46.0 (13.53)	White UK (55.6%); African-Caribbean (11.1%); Thai (11.1%); Kurdish (11.1%); Indian (11.1%)
**Joung et al. (2025)**	Korea	Qualitative; Focused etnography	Care farming	Schizophrenia (NR)	~16 weeks	8 sessions 1 ×/2 weeks × 2 h	6	2/4	39.0 (11.0)	NR
**Iancu et al. (2014)**	Netherlands	Qualitative; Not reported	Care farming	Common (depressive, anxiety) and severe (schizophrenia, personality) mental disorders (NR)	NR	Daily × NR	26	16/10	42.5 (11.6)	NR
**Pedersen et al. (2012a)**	Norway	Qualitative; Individual thematic interviews	Care farming	Depression (MINI and/or BDI)	12 weeks	2×/week × NR	8	1/7	37.62 (11.58)	NR
**Leighton et al. (2021)**	Canada	Qualitative; Thematic Analysis	Integrating Talking Therapies	Dual PTS-substance use disorder diagnosis (NR)	6 h	1× (day) × 6 h	6	3/3	NR	NR
**Cooley et al. (2021)**	UK	Qualitative; Ethnographical study guided by an experiential pragmatist approach	Exercise Focused	Depression; anxiety; PTSD; psychosis; schizophrenia (NR)	23 weeks during a year	23 walks over 23 weeks; 7 current patients (3 first-year, 4 with 1–7 years’ prior attendance) + 22 historical cases	29	15/14	52.14 (6.20)	White British (85.7%); South Asian (14.3%)

**Table 2:** Clinical symptom outcomes for intervention and comparison groups across included quantitative studies, showing pre-post changes on measures of global psychopathology, mood, anxiety, affect, and healthcare utilisation. BPRS = Brief Psychiatric Rating Scale; PANSS = Positive and Negative Syndrome Scale; CGI = Clinical Global Impression; CGI-S = Clinical Global Impression–Severity; CGI-I = Clinical Global Impression–Improvement; DASS-21 = Depression Anxiety Stress Scale-21; BDI-IA = Beck Depression Inventory–First Amended; STAI = State–Trait Anxiety Inventory; STAI-SS = State–Trait Anxiety Inventory–State Subscale; MADRS = Montgomery–Åsberg Depression Rating Scale; PHQ-9 = Patient Health Questionnaire-9; PANAS = Positive and Negative Affect Schedule; PANAS-SF = Positive and Negative Affect Schedule–Short Form; POMS = Profile of Mood States; HDRS-21 = 21-item Hamilton Depression Rating Scale; IG = intervention group; CG = control (comparison) group; PA = Positive Affect; NA = Negative Affect; NR = not reported; NS = not significant; FU = follow-up.

Author(s)	NBI	Diagnosis (Criteria)	Outcome Tool	Subscale/Measure	Group	Statistical Test	Pre-Mean (SD)	Post Mean (SD)	*p* Value/Notes
**He et al.**	Horticulture	Schizophrenia (ICD-10)	BPRS	Total	IG/CG	Paired *t*-test	IG: 35.50 (6.27) CG: 37.86 (4.38)	IG: 31.83 (4.70) CG: 37.38 (4.62)	0.000
**Oh et al.**	Horticulture	Schizophrenia (NR)	BPRS	Total	IG/CG	Paired *t*-test	IG: 26.40 (9.83) CG: 20.77 (11.79)	IG: 19.53 (11.12) CG: 19.77 (11.56)	0.000
			PANSS	Total	IG/CG	Paired *t*-test	IG: 102.00 (27.69) CG: 89.46 (32.10)	IG: 84.33 (27.63) CG: 89.46 (32.90)	0.000
**Zhu et al.**	Horticulture	Schizophrenia (ICD-10)	PANSS	Total	IG/CG	*T*-test; ANOVA; Repeated-measures ANOVA	IG: 48.1 (5.4) CG: 48.3 (5.8)	IG—After 4 weeks: 41.7 (4.6) CG—After 4 weeks: 45.3 (4.8) IG—After 12 weeks: 37.4 (3.4) CG—After 12 weeks: 41.7 (4.5)	<0.001
**Währborg et al.**	Horticulture	Exhaustion syndrome; depression; PTSD 9 ICD-10)	Sick-leave status	Bed days (somatic, psychiatric, total)	IG/CG	ANOVA; Regression analysis	NR	NR	All *p* < 0.05
			Healthcare consumption	Somatic, psychiatric, primary, total	IG/CG	ANOVA; Regression analysis	IG: 28.7 CG: 18.3	IG: 24.1 CG: 16.8	SH NS; PSH NS; PRH < 0.05; OT < 0.05
**Trkulja et al.**	Horticulture	Adjustment disorder, reaction to severe stress, anxiety, depression (ICD-10)	CGI	Severity (CGI-S), Improvement (CGI-I)	IG/CG	ANOVA (2 × 2 mixed)	IG: 4.20 (1.08) CG: 3.75 (1.14)	IG: 2.47 (1.19) CG: 3.75 (1.14)	0.001
**Siu et al.**	Horticulture	Schizophrenia; Other Psychiatric Illness (NR)	DASS-21	Anxiety, Stress	IG/CG	Repeated-measures ANOVA	Anxiety: IG 0.59 (0.55), CG 0.71 (0.47); Stress: IG 0.73 (0.66), CG 0.85 (0.48)	Anxiety FU: IG 0.72 (0.59), CG 0.84 (0.68); Stress FU: IG 0.87 (0.68), CG 0.96 (0.66)	0.29 (Anxiety); 0.36 (Stress)
**Kam et al.**	Horticulture	Schizophrenia spectrum; bipolar disorder; major depression (NR)	DASS-21	Depression, Anxiety, Stress	IG/CG	*T*-test	Depression: 14.6 (9.1); Anxiety: 15.0 (7.8); Stress: 12.6 (7.7)	Depression: −9.20 (9.15); Anxiety: −9.0 (7.26); Stress: −6.00 (5.33)	Depression 0.04; Anxiety 0.01; Stress 0.05
**Pedersen et al.**	Care farming	Major depressive disorder (BDI-IA ≥ 14; DSM-IV major depression—MINI in subsample)	BDI-IA	Total	IG/CG	Paired *t*-test	IG: 26.5 (9.2); CG: 32.0 (7.2)	IG: 17.3 (12.6); CG: 28.2 (11.0)	IG 0.003; CG NS
			STAI-SS	Total	IG/CG	Paired *t*-test	IG: 55.0 (10.6); CG: 60.5 (8.6)	IG: 49.4 (13.9); CG: 55.5 (13.1)	IG 0.059; CG NS
**Shimizu et al.**	Care farming	Schizophrenia (DSM-IV-TR)	STAI	A-Trait, A-State	IG/CG	Mann–Whitney U-test	AT: IG: 45.33 (3.68), CG 46.64 (2.10); AS: IG: 40.75 (3.98), CG: 40.00 (3.75)	AT IG 44.00 (2.44), CG 42.55 (2.67). AS IG 35.67 (2.37), CG 41.18 (3.09)	AT 0.370; AS 0.198
**Berget et al.**	Care farming	Schizophrenia/schizotypal; affective; anxiety/stress-related; personality disorders (ICD-10)	STAI	Total	IG	NA	51.91 (2.33)	50.50 (2.35)	NR
			BDI	Total	IG	NA	20.44 (1.96)	16.91 (1.95)	NR
**Pedersen et al.**	Care farming	Depression (DSM-IV and BDI-IA)	BDI-IA	Total	IG	Spearman’s ρ	25.9 (2.8)	19.1 (3.9)	Milking *p* = 0.02; Moving *p* = 0.03
			STAI-SS	Total	IG	Spearman’s ρ	55.3 (2.7)	49.6 (4.1)	Milking *p* = 0.01; Moving *p* = 0.01; Grooming *p* = 0.02; Dialogue *p* = 0.05
**Walter et al.**	Exercise Focused	Major depressive disorder (MINI-7)	MADRS	Total	IG (Surf)/CG (Hike)	Multilevel modelling	IG 25.92 (8.23); CG 28.02 (8.56)	Post: IG 18.62 (11.35), CG 21.46 (11.43); 3-month: IG 15.63 (11.79), CG 20.13 (13.45)	*p* < 0.001 (time effect)
			PHQ-9	Total	IG/CG	Multilevel modelling	IG 15.96 (4.78); CG 18.19 (4.78)	Post: IG 10.72 (6.61), CG 13.63 (6.98); 3-month: IG 8.88 (6.22), CG 12.13 (7.08)	*p* < 0.001
**Keenan et al.**	Exercise Focused	Anxiety; depression (NR)	PANAS	Positive Affect (PA), Negative Affect (NA)	IG/CG	*T*-test	PA IG: 15.68 (2.23) CG: 15.12 (2.05) NA IG: 42.60 (4.37), CG: 43.76 (4.49)	PA: IG: 30.40 (4.01), CG: 17.88 (4.28) NA: IG: 29.68 (5.66) CG: 40.24 (5.95) PA Follow-up: IG: 36.88 (2.14) CG: 28.04 (3.01) NA Follow-up: IG: 36.60 (1.68) CG: 20.84 (4.03)	<0.05
**Barton et al.**	Exercise Focused	Anxiety; depression (NR)	POMS (Short Form)	Total Mood Disturbance (TMD)	IG/CG	Paired *t*-test	Green Exercise: 154.3 (24.6); Swimming: 155.4 (26.4); Social: 142.4 (26.2)	Green Exercise: 143.1 (24.6); Swimming: 142.1 (21.1); Social: 134.4 (21.2)	<0.0001 (all)
**Iwata et al.**	Exercise Focused	Depression; bipolar disorder; anxiety disorder; other (NR)	HDRS-21	Total	IG	—	11.84 (NR)	5.98 (NR)	—
			PANAS	PA, NA	IG	ANOVA	PA Week 8: 33.6 (12.1), Week 13: 35.4 (12.5); NA Week 8: 21.1 (9.7), Week 13: 21.9 (9.7)	PA Week 8: 39.2 (12.1), Week 13: 40.2 (9.0); NA Week 8: 16.7 (6.7), Week 13: 17.7 (10.7)	PA W8 *p* < 0.001; W13 NS; NA W8 *p* < 0.05; W13 NS
			BDI	Total	IG	—	22.86 (NR)	14.93 (NR)	—
**Müller et al.**	Integrating Alternative Therapies	Depression (NR)	PANAS-SF	PA, NA	IG/CG	Linear mixed models	PA: IG 2.46, CG: 2.50; NA: IG 2.98, CG: 2.88	PA: IG 3.46, CG 3.18; NA: IG 1.98, CG 2.31	PA 0.013; NA 0.005
			PHQ-9	Total	IG/CG	Linear mixed models	IG 12.32; CG 12.31	IG 7.38; CG 8.46	0.15

## Data Availability

The data that support the findings of this study are available from the corresponding author upon reasonable request.

## Supplementary Materials

The following supporting information can be downloaded at: https://www.mdpi.com/article/10.3390/bs16060974/s1.
