# Supplementary material for: Nature-Based Interventions for Individuals with Psychiatric Disorders: A Mixed Methods Systematic Review with Random-Effects Meta-Analysis of Mental Health and Functional Outcomes
Source: Behav Sci (Basel). 2026 Jun 11;16(6):974. doi: 10.3390/bs16060974 (PMC13295733; doi:10.3390/bs16060974)
Supplement: Supplementary file 1 [file behavsci-16-00974-s001.zip › behavsci-4275268-supplementary.pdf]

Nature-Based Interventions for Individuals with Psychiatric Disorders: A Mixed Methods Review of Mental Health and Functional Outcomes

Supplementary Material

**File S1**	**Search terms and search strategies**	Pp 2–18
	**Prisma flow chart**	Pp 25
**File S2**	**Intervention and comparator details**<br>*Table S1*. Intervention and control group details of the examined studies	Pp 26-30
**File S3**	**Quantitative outcome tables**<br>*Table S2.* Quality of life and wellbeing outcomes of the examined studies.<br>*Table S3.* Psychosocial outcomes of the examined studies.<br>*Table S4.* Functional outcomes of the examined studies.<br>*Table S5.* Physical health outcomes of the examined studies	Pp 31-37
**File S3**	**Qualitative synthesis: themes and exemplar quotes**<br>*Table S6. Themes, subthemes, and exemplar quotes (S1–S26)*	Pp 38-40
**File S4**	**Quality appraisal**	Pp 41-45

File S1. Search terms and search strategy

Search strategy

1 exp green space/
2 green space?.ti,ab,kw.
3 greenspace?.ti,ab,kw.
4 wilderness therap*.ti,ab,kw.
5 wilderness.ti,ab,kw.
6 exp horticultural therapy/
7 horticultur* therap*.ti,ab,kw.
8 horticultur*.ti,ab,kw.
9 floricultcur*.ti,ab,kw.
10 exp Animal Assisted Therapy/
11 (animal? adj3 therap*).ti,ab,kw.
12 natural environment?.ti,ab,kw.
13 nature experience?.ti,ab,kw.
14 forest bathing.ti,ab,kw.
15 shinrin yoku.ti,ab,kw.
16 ecotherap*.ti,ab,kw.
17 care farm*.ti,ab,kw.
18 (therap* adj4 farm*).ti,ab,kw.
19 therapeutic space?.ti,ab,kw.
20 exp Mental Disorders/ or exp Mental Health/
21 (mental adj2 disorder?).ti,ab,kw.
22 exp Psychotic Disorders/ or exp Mental Disorders/ or exp Bipolar Disorder/ or exp Substance-Related Disorders/ or exp Schizophrenia/
23 psychiatric illness.ti,ab,kw.
24 psychiatric disorder?.ti,ab,kw.
25 exp Mood Disorders/
26 mood disorder?.ti,ab,kw.
27 affective disorder?.ti,ab,kw.
28 exp Anxiety Disorders/
29 anxiety disorder?.ti,ab,kw.
30 exp Psychotic Disorders/
31 psychosis.ti,ab,kw.

32 exp Personality Disorders/

33 personality disorder?.ti,ab,kw.

34 schizophrenia spectrum disorder?.ti,ab,kw.

35 exp Depressive Disorder/

36 depressive disorder?.ti,ab,kw.

37 psychiatric condition?.ti,ab,kw.

38 psychological disorder?.ti,ab,kw.

39 emotional disorder?.ti,ab,kw.

40 exp Rehabilitation Centers/ or exp Psychiatric Rehabilitation/ or exp Rehabilitation/

41 rehabilitat*.ti,ab,kw.

42 recover*.ti,ab,kw.

43 reintegration?.ti,ab,kw.

44 restorative therap*.ti,ab,kw.

45 neurorehabilitat*.ti,ab,kw.

46 occupational therap*.ti,ab,kw.

47 1 or 2 or 3 or 4 or 5 or 6 or 7 or 8 or 9 or 10 or 11 or 12 or 13 or 14 or 15 or 16 or 17 or 18 or 19

48 20 or 21 or 22 or 23 or 24 or 25 or 26 or 27 or 28 or 29 or 30 or 31 or 32 or 33 or 34 or 35 or 36 or 37 or 38 or 39

49 40 or 41 or 42 or 43 or 44 or 45 or 46

50 47 and 48 and 49

EMBASE (OVID)

**1**	exp green space/	0
**2**	green space?.ti,ab,kw.	3273
**3**	greenspace?.ti,ab,kw.	940
**4**	wilderness therap*.ti,ab,kw.	25
**5**	wilderness.ti,ab,kw.	1814
**6**	exp horticultural therapy/	208
**7**	horticultur* therap*.ti,ab,kw.	202
**8**	horticultur*.ti,ab,kw.	5215
**9**	floricultcur*.ti,ab,kw.	0
**10**	exp Animal Assisted Therapy/	1683
**11**	(animal? adj3 therap*).ti,ab,kw.	6724
**12**	natural environment?.ti,ab,kw.	16944
**13**	nature experience?.ti,ab,kw.	135

14 forest bathing.ti,ab,kw. 114

15 shinrin yoku.ti,ab,kw. 64

16 ecotherap*.ti,ab,kw. 29

17 care farm*.ti,ab,kw. 96

18 (therap* adj4 farm*).ti,ab,kw. 326

19 therapeutic space?.ti,ab,kw. 303

20 exp Mental Disorders/ or exp Mental Health/ 3010696

21 (mental adj2 disorder?).ti,ab,kw. 79760

22 exp Psychotic Disorders/ or exp Mental Disorders/ or exp Bipolar Disorder/ or exp Substance-Related Disorders/ or exp Schizophrenia/ 2884994

23 psychiatric illness.ti,ab,kw. 12520

24 psychiatric disorder?.ti,ab,kw. 81506

25 exp Mood Disorders/ 730793

26 mood disorder?.ti,ab,kw. 36930

27 affective disorder?.ti,ab,kw. 27077

28 exp Anxiety Disorders/ 347657

29 anxiety disorder?.ti,ab,kw. 61737

30 exp Psychotic Disorders/ 342457

31 psychosis.ti,ab,kw. 75470

32 exp Personality Disorders/ 72018

33 personality disorder?.ti,ab,kw. 33044

34 schizophrenia spectrum disorder?.ti,ab,kw. 5699

35 exp Depressive Disorder/ 679073

36 depressive disorder?.ti,ab,kw. 71963

37 psychiatric condition?.ti,ab,kw. 12203

38 psychological disorder?.ti,ab,kw. 7547

39 emotional disorder?.ti,ab,kw. 5392

40 exp Rehabilitation Centers/ or exp Psychiatric Rehabilitation/ or exp Rehabilitation/ 533540

41 rehabilitat*.ti,ab,kw. 330570

42 recover*.ti,ab,kw. 1115680

43 reintegration?.ti,ab,kw. 5910

44 restorative therap*.ti,ab,kw. 773

45 neurorehabilitat*.ti,ab,kw. 8163

46 occupational therap*.ti,ab,kw. 28019

47 1 or 2 or 3 or 4 or 5 or 6 or 7 or 8 or 9 or 10 or 11 or 12 or 13 or 14 or 15 or 16 or 17 or 18 or 19 36508

48 20 or 21 or 22 or 23 or 24 or 25 or 26 or 27 or 28 or 29 or 30 or 31 or 32 or 33 or 34 or 35 or 36 or 37 or 38 or 39 3048432

49 40 or 41 or 42 or 43 or 44 or 45 or 46 1772677

50 47 and 48 and 49 675

MEDLINE (OVID)

1 exp green space/ 2882

2 green space?.ti,ab,kw. 3201

3 greenspace?.ti,ab,kw. 907

4 wilderness therap*.ti,ab,kw. 21

5 wilderness.ti,ab,kw. 1384

6 exp horticultural therapy/ 104

7 horticultur* therap*.ti,ab,kw. 154

8 horticultur*.ti,ab,kw. 5651

9 floricultcur*.ti,ab,kw. 0

10 exp Animal Assisted Therapy/ 891

11 (animal? adj3 therap*).ti,ab,kw. 4983

12 natural environment?.ti,ab,kw. 15809

13 nature experience?.ti,ab,kw. 153

14 forest bathing.ti,ab,kw. 104

15 shinrin yoku.ti,ab,kw. 60

16 ecotherap*.ti,ab,kw. 20

17 care farm*.ti,ab,kw. 91

18 (therap* adj4 farm*).ti,ab,kw. 227

19 therapeutic space?.ti,ab,kw. 176

20 exp Mental Disorders/ or exp Mental Health/ 1558184

21 (mental adj2 disorder?).ti,ab,kw. 61848

22 exp Psychotic Disorders/ or exp Mental Disorders/ or exp Bipolar Disorder/ or exp Substance-Related Disorders/ or exp Schizophrenia/ 1530574

23 psychiatric illness.ti,ab,kw. 8185

24 psychiatric disorder?.ti,ab,kw. 55745

25 exp Mood Disorders/ 175546

26 mood disorder?.ti,ab,kw. 23618

27 affective disorder?.ti,ab,kw. 19132

28 exp Anxiety Disorders/ 94729

29 anxiety disorder?.ti,ab,kw. 43796

30 exp Psychotic Disorders/ 60496

31 psychosis.ti,ab,kw. 49074

32 exp Personality Disorders/ 46211

33 personality disorder?.ti,ab,kw. 24235

34 schizophrenia spectrum disorder?.ti,ab,kw. 4110

35 exp Depressive Disorder/ 126174

36 depressive disorder?.ti,ab,kw. 51313

37 psychiatric condition?.ti,ab,kw. 8159

38 psychological disorder?.ti,ab,kw. 5367

39 emotional disorder?.ti,ab,kw. 3949

40 exp Rehabilitation Centers/ or exp Psychiatric Rehabilitation/ or exp Rehabilitation/ 380237

41 rehabilitat*.ti,ab,kw. 237657

42 recover*.ti,ab,kw. 865931

43 reintegration?.ti,ab,kw. 4246

44 restorative therap*.ti,ab,kw. 578

45 neurorehabilitat*.ti,ab,kw. 5201

46 occupational therap*.ti,ab,kw. 19840

47 1 or 2 or 3 or 4 or 5 or 6 or 7 or 8 or 9 or 10 or 11 or 12 or 13 or 14 or 15 or 16 or 17 or 18 or 19 34601

48 20 or 21 or 22 or 23 or 24 or 25 or 26 or 27 or 28 or 29 or 30 or 31 or 32 or 33 or 34 or 35 or 36 or 37 or 38 or 39 1670122

49 40 or 41 or 42 or 43 or 44 or 45 or 46 1381548

50 47 and 48 and 49 573

PsycINFO (OVID)

exp green space/ 263

2 green space?.ti,ab. 561

3 greenspace?.ti,ab. 149

4 wilderness therap*.ti,ab.156

5 wilderness.ti,ab.968

6 horticultur* therap*.ti,ab. 126

7 horticultur*.ti,ab. 522

8 floricultcur*.ti,ab. 0

9 exp Animal Assisted Therapy/ 1582

10 (animal? adj3 therap*).ti,ab. 1055

11 natural environment?.ti,ab. 4827

12 nature experience?.ti,ab. 217

13 forest bathing.ti,ab. 18

14 shinrin yoku.ti,ab. 9

15 ecotherap*.ti,ab. 78

16 care farm*.ti,ab. 49

17 (therap* adj4 farm*).ti,ab. 41

18 therapeutic space?.ti,ab. 444

19 exp Mental Disorders/ or exp Mental Health/ 1208342

20 (mental adj2 disorder?).ti,ab. 64263

21 exp Psychotic Disorders/ or exp Mental Disorders/ or exp Bipolar Disorder/ or exp Substance-Related Disorders/ or exp Schizophrenia/ 1141000

22 psychiatric illness.ti,ab. 6808

23 psychiatric disorder?.ti,ab. 43881

24 exp Mood Disorders/ 188171

25 mood disorder?.ti,ab. 18236

26 affective disorder?.ti,ab. 18311

27 exp Anxiety Disorders/ 45108

28 anxiety disorder?.ti,ab. 40961

29 exp Psychotic Disorders/ 134024

30 psychosis.ti,ab. 46035

31 exp Personality Disorders/ 33294

32 personality disorder?.ti,ab. 35851

33 schizophrenia spectrum disorder?.ti,ab. 3700

34 exp Depressive Disorder/ 188171

35 depressive disorder?.ti,ab. 39958

36 psychiatric condition?.ti,ab. 6475

37 psychological disorder?.ti,ab. 5466

38 emotional disorder?.ti,ab. 4451

39 exp Rehabilitation Centers/ or exp Psychiatric Rehabilitation/ or exp Rehabilitation/ 64249

40 rehabilitat*.ti,ab. 67941

41 recover*.ti,ab. 100427

42 reintegration?.ti,ab. 4285

43 restorative therap*.ti,ab. 87

44 neurorehabilitat*.ti,ab. 1882

45 occupational therap*.ti,ab. 12617

46 1 or 2 or 3 or 4 or 5 or 6 or 7 or 8 or 9 or 10 or 11 or 12 or 13 or 14 or 15 or 16 or 17 or 18 9799

47 19 or 20 or 21 or 22 or 23 or 24 or 25 or 26 or 27 or 28 or 29 or 30 or 31 or 32 or 33 or 34 or 35 or 36 or 37 or 38 1254727

48 39 or 40 or 41 or 42 or 43 or 44 or 45 198408

49 46 and 47 and 48 228

Global Health

1 exp green space/ 0

2 green space?.ti,ab. 2116

3 greenspace?.ti,ab. 493

4 wilderness therap*.ti,ab. 11

5 wilderness.ti,ab. 371

6 horticultur* therap*.ti,ab. 226

7 horticultur*.ti,ab. 3668

8 floricultcur*.ti,ab. 0

9 exp Animal Assisted Therapy/ 531

10 (animal? adj3 therap*).ti,ab. 955

11 natural environment?.ti,ab. 3740

12 nature experience?.ti,ab. 53

13 forest bathing.ti,ab. 74

14 shinrin yoku.ti,ab. 36

15 ecotherap*.ti,ab. 10

16 care farm*.ti,ab. 58

17 (therap* adj4 farm*).ti,ab. 139

18 therapeutic space?.ti,ab. 11

19 exp Mental Disorders/ or exp Mental Health/ 128145

20 (mental adj2 disorder?).ti,ab. 9405

21 exp Psychotic Disorders/ or exp Mental Disorders/ or exp Bipolar Disorder/ or exp Substance-Related Disorders/ or exp Schizophrenia/ 112495

22 psychiatric illness.ti,ab. 772

23 psychiatric disorder?.ti,ab. 4983
24 exp Mood Disorders/ 0
25 mood disorder?.ti,ab. 2075
26 affective disorder?.ti,ab. 908
27 exp Anxiety Disorders/ 0
28 anxiety disorder?.ti,ab. 3996
29 exp Psychotic Disorders/ 10487
30 psychosis.ti,ab. 2048
31 exp Personality Disorders/ 0
32 personality disorder?.ti,ab. 902
33 schizophrenia spectrum disorder?.ti,ab. 122
34 exp Depressive Disorder/ 0
35 depressive disorder?.ti,ab. 3924
36 psychiatric condition?.ti,ab. 623
37 psychological disorder?.ti,ab. 939
38 emotional disorder?.ti,ab. 384
39 exp Rehabilitation Centers/ or exp Psychiatric Rehabilitation/ or exp Rehabilitation/ 2660
40 rehabilitat*.ti,ab. 14343
41 recover*.ti,ab. 150155
42 reintegration?.ti,ab. 456
43 restorative therap*.ti,ab. 20
44 neurorehabilitat*.ti,ab. 74
45 occupational therap*.ti,ab. 668
46 1 or 2 or 3 or 4 or 5 or 6 or 7 or 8 or 9 or 10 or 11 or 12 or 13 or 14 or 15 or 16 or 17 or 18 11710
47 19 or 20 or 21 or 22 or 23 or 24 or 25 or 26 or 27 or 28 or 29 or 30 or 31 or 32 or 33 or 34 or 35 or 36 or 37 or 38 132061
48 39 or 40 or 41 or 42 or 43 or 44 or 45 163828
49 46 and 47 and 48 90

Cochrane Central Register of Controlled Trials

1 exp green space/ (55)
2 green space?.ti,ab,kw. (55)

3 greenspace?.ti,ab,kw. (18)

4 wilderness therap*.ti,ab,kw. (2)

5 wilderness.ti,ab,kw. (55)

6 exp horticultural therapy/ (2)

7 horticultur* therap*.ti,ab,kw. (102)

8 horticultur*.ti,ab,kw. (173)

9 floricultcur*.ti,ab,kw. (0)

10 exp Animal Assisted Therapy/ (177)

11 (animal? adj3 therap*).ti,ab,kw. (440)

12 natural environment?.ti,ab,kw. (2040)

13 nature experience?.ti,ab,kw. (7719)

14 forest bathing.ti,ab,kw. (37)

15 shinrin yoku.ti,ab,kw. (13)

16 ecotherap*.ti,ab,kw. (0)

17 care farm*.ti,ab,kw. (295)

18 (therap* adj4 farm*).ti,ab,kw. (27)

19 therapeutic space?.ti,ab,kw. (2390)

20 exp Mental Disorders/ or exp Mental Health/ (43939)

21 (mental adj2 disorder?).ti,ab,kw. (15987)

22 exp Psychotic Disorders/ or exp Mental Disorders/ or exp Bipolar Disorder/ or exp Substance-Related Disorders/ or exp Schizophrenia/ (43592)

23 psychiatric illness.ti,ab,kw. (1059)

24 psychiatric disorder?.ti,ab,kw. (22248)

25 exp Mood Disorders/ (4272)

26 mood disorder?.ti,ab,kw. (13283)

27 affective disorder?.ti,ab,kw. (25653)

28 exp Anxiety Disorders/ (16203)

29 anxiety disorder?.ti,ab,kw. (34267)

30 exp Psychotic Disorders/ (5046)

31 psychosis.ti,ab,kw. (7781)

32 exp Personality Disorders/ (3406)

33 personality disorder?.ti,ab,kw. (23231)

34 schizophrenia spectrum disorder?.ti,ab,kw. (1036)

35 exp Depressive Disorder/ (22154)

36 depressive disorder?.ti,ab,kw. (51898)

37 psychiatric condition?.ti,ab,kw. (9246)

38 psychological disorder?.ti,ab,kw. (46089)

39 emotional disorder?.ti,ab,kw. (13863)

40 exp Rehabilitation Centers/ or exp Psychiatric Rehabilitation/ or exp Rehabilitation/ (2953)

41 rehabilitat*.ti,ab,kw. (68184)

42 recover*.ti,ab,kw. (92260)

43 reintegration?.ti,ab,kw. (645)

44 restorative therap*.ti,ab,kw. (11538)

45 neurorehabilitat*.ti,ab,kw. (1065)

46 occupational therap*.ti,ab,kw. (9353)

47 1 or 2 or 3 or 4 or 5 or 6 or 7 or 8 or 9 or 10 or 11 or 12 or 13 or 14 or 15 or 16 or 17 or 18 or 19 (12248)

48 20 or 21 or 22 or 23 or 24 or 25 or 26 or 27 or 28 or 29 or 30 or 31 or 32 or 33 or 34 or 35 or 36 or 37 or 38 or 39 (158941)

49 40 or 41 or 42 or 43 or 44 or 45 or 46 (170360)

50 47 and 48 and 49 (354)

Greenfile

#	Query	Limiters/Expanders	Results
S48	S45 AND S46 AND S47	Expanders - Apply equivalent subjects<br>Search modes - Proximity	45
S47	S39 OR S40 OR S41 OR S42 OR S43 OR S44	Expanders - Apply equivalent subjects<br>Search modes - Proximity	58,123
S46	S18 OR S19 OR S20 OR S21 OR S22 OR S23 OR S24 OR S25 OR S26 OR S27 OR S28 OR S29 OR S30 OR S31 OR S32 OR S33 OR S34 OR S35 OR S36 OR S37 OR S38	Expanders - Apply equivalent subjects<br>Search modes - Proximity	2,910
S45	S1 OR S2 OR S3 OR S4 OR S5 OR S6 OR S7 OR S8 OR S9 OR	Expanders - Apply equivalent subjects<br>Search modes - Proximity	83,398

	S10 OR S11 OR S12 OR S13 OR S14 OR S15 OR S16 OR S17		
S44	occupational therap*	Expanders - Apply equivalent subjects<br>Search modes - Find all my search terms	337
S43	neurorehabilitat*	Expanders - Apply equivalent subjects<br>Search modes - Find all my search terms	2
S42	restorative therap*	Expanders - Apply equivalent subjects<br>Search modes - Find all my search terms	29
S41	reintegration#	Expanders - Apply equivalent subjects<br>Search modes - Find all my search terms	51
S40	recover*	Expanders - Apply equivalent subjects<br>Search modes - Find all my search terms	54,703
S39	rehabilitat*	Expanders - Apply equivalent subjects<br>Search modes - Find all my search terms	3,348
S38	emotional disorder#	Expanders - Apply equivalent subjects<br>Search modes - Find all my search terms	76
S37	psychological disorder#	Expanders - Apply equivalent subjects	233

		Search modes - Find all my search terms	
S36	psychiatric condition#	Expanders - Apply equivalent subjects<br>Search modes - Find all my search terms	43
S35	depressive disorder#	Expanders - Apply equivalent subjects<br>Search modes - Find all my search terms	242
S34	schizophrenia spectrum disorder#	Expanders - Apply equivalent subjects<br>Search modes - Find all my search terms	31
S33	personality disorder#	Expanders - Apply equivalent subjects<br>Search modes - Find all my search terms	24
S32	psychosis	Expanders - Apply equivalent subjects<br>Search modes - Find all my search terms	34
S31	psychotic disorder#	Expanders - Apply equivalent subjects<br>Search modes - Find all my search terms	19
S30	anxiety disorder#	Expanders - Apply equivalent subjects<br>Search modes - Find all my search terms	211
S29	affective disorder#	Expanders - Apply equivalent subjects	42

		Search modes - Find all my search terms	
S28	mood disorder#	Expanders - Apply equivalent subjects<br>Search modes - Find all my search terms	62
S27	psychiatric disorder#	Expanders - Apply equivalent subjects<br>Search modes - Find all my search terms	253
S26	psychiatric illness	Expanders - Apply equivalent subjects<br>Search modes - Find all my search terms	153
S25	schizophren*	Expanders - Apply equivalent subjects<br>Search modes - Find all my search terms	109
S24	schizophrenia	Expanders - Apply equivalent subjects<br>Search modes - Find all my search terms	103
S23	substance disorder#	Expanders - Apply equivalent subjects<br>Search modes - Find all my search terms	371
S22	bipolar disorder#	Expanders - Apply equivalent subjects<br>Search modes - Find all my search terms	36
S21	psychotic disorder#	Expanders - Apply equivalent subjects	19

		Search modes - Find all my search terms	
S20	mental N3 disorder#	Expanders - Apply equivalent subjects<br>Search modes - Find all my search terms	221
S19	mental disorder#	Expanders - Apply equivalent subjects<br>Search modes - Find all my search terms	518
S18	mental health	Expanders - Apply equivalent subjects<br>Search modes - Find all my search terms	2,112
S17	therapeutic space#	Expanders - Apply equivalent subjects<br>Search modes - Find all my search terms	56
S16	therap* N4 farm*	Expanders - Apply equivalent subjects<br>Search modes - Find all my search terms	5
S15	care farm*	Expanders - Apply equivalent subjects<br>Search modes - Find all my search terms	455
S14	ecotherap*	Expanders - Apply equivalent subjects<br>Search modes - Find all my search terms	24
S13	shinrin-yoku	Expanders - Apply equivalent subjects	11

		Search modes - Find all my search terms	
S12	forest bathing	Expanders - Apply equivalent subjects<br>Search modes - Find all my search terms	20
S11	natur* experience#	Expanders - Apply equivalent subjects<br>Search modes - Find all my search terms	9,157
S10	natural environment#	Expanders - Apply equivalent subjects<br>Search modes - Find all my search terms	65,937
S9	animal# N3 therap*	Expanders - Apply equivalent subjects<br>Search modes - Find all my search terms	32
S8	animal assisted therapy	Expanders - Apply equivalent subjects<br>Search modes - Find all my search terms	8
S7	floricultur*	Expanders - Apply equivalent subjects<br>Search modes - Find all my search terms	188
S6	horticultur*	Expanders - Apply equivalent subjects<br>Search modes - Find all my search terms	5,899
S5	horticultur* therap*	Expanders - Apply equivalent subjects	50

		Search modes - Find all my search terms	
S4	wilderness	Expanders - Apply equivalent subjects<br>Search modes - Find all my search terms	2,905
S3	wilderness therap*	Expanders - Apply equivalent subjects<br>Search modes - Find all my search terms	12
S2	greenspace#	Expanders - Apply equivalent subjects<br>Search modes - Find all my search terms	633
S1	green space#	Expanders - Apply equivalent subjects<br>Search modes - Find all my search terms	5,116

Web of Science

1.

(ALL=("green space$")
 OR ALL=(greenspace$)
 OR ALL=("wilderness therap*")
 OR ALL=(wilderness)
 OR ALL=("horticultur* therap*")
 OR ALL=(horticultur*)
 OR ALL=(floricultur*)
 OR ALL=("animal assisted therapy")
 OR ALL=("animal$ NEAR/3 therap*")
 OR ALL=("natural environment$")
 OR ALL=("nature experience$")
 OR ALL=("forest bathing")

OR ALL=("shinrin yoku")
OR ALL=(ecotherap*)
OR ALL=("care farm*")
OR ALL=("therap* NEAR/4 farm*")
OR ALL=("therapeutic space$")
)
NOT (SILOID==("PPRN"))

Date run: Wed 09 Oct 2024, 13:51 (GMT+0200, Central European Summer Time)
Results: 299,166

2.
(ALL=("mental NEAR/2 disorder$")
OR ALL=("mental health")
OR ALL=("psychotic disorder$")
OR ALL=("bipolar disorder$")
OR ALL=("substance disorder$")
OR ALL=("mental disorder$")
OR ALL=(schizophrenia)
OR ALL=(schizophren*)
OR ALL=("psychiatric illness")
OR ALL=("psychiatric disorder$")
OR ALL=("mood disorder$")
OR ALL=("affective disorder$")
OR ALL=("anxiety disorder$")
OR ALL=("psychotic disorder$")
OR ALL=(psychosis)
OR ALL=("personality disorder$")
OR ALL=("schizophrenia spectrum disorder$")
OR ALL=("depressive disorder$")
OR ALL=("psychiatric condition$")
OR ALL=("psychological disorder$")
OR ALL=("emotional disorder$")
)
NOT (SILOID==("PPRN"))

Date run: Wed 09 Oct 2024, 13:55:55 (GMT+0200, Central European Summer Time)

Results: 1,066,845

3.

(ALL=(rehabilitat*)

OR ALL=(recover*)

OR ALL=(reintegration$)

OR ALL=("restorative therap*")

OR ALL=(neurorehabilitat*)

OR ALL=("occupational therap*")

)

NOT (SILOID==("PPRN"))

Date run: Wed 09 Oct 2024, 13:56:52 (GMT+0200, Central European Summer Time)

Results: 2,136,040

4.

#3 AND #2 AND #1

Date run: Wed 09 Oct 2024, 13:57:06 (GMT+0200, Central European Summer Time)

Results: 481

Scopus

1. TI=("green space$") OR AB=("green space$") OR AK=("green space$")
   Date run: Fri 11 Oct 2024, 21:07:11 (GMT+0200, Central European Summer Time)
   Results: 11,844

2. TI=("greenspace$") OR AB=("greenspace$") OR AK=("greenspace$")
   Date run: Fri 11 Oct 2024, 21:07:11 (GMT+0200)
   Results: 2,181

3. TI=("wilderness therap*") OR AB=("wilderness therap*") OR AK=("wilderness therap*")

Date run: Fri 11 Oct 2024, 21:07:11 (GMT+0200)

Results: 101

4. TI=("wilderness") OR AB=("wilderness") OR AK=("wilderness")

Date run: Fri 11 Oct 2024, 21:07:12 (GMT+0200)

Results: 8,959

5. TI=("horticultur* therap*") OR AB=("horticultur* therap*") OR AK=("horticultur* therap*")

Date run: Fri 11 Oct 2024, 21:07:12 (GMT+0200)

Results: 476

6. TI=(horticultur*) OR AB=(horticultur*) OR AK=(horticultur*)

Date run: Fri 11 Oct 2024, 21:07:13 (GMT+0200)

Results: 27,670

7. TI=(floricultur*) OR AB=(floricultur*) OR AK=(floricultur*)

Date run: Fri 11 Oct 2024, 21:07:13 (GMT+0200)

Results: 2,057

8. TI=(animal$ NEAR/3 therap*) OR AB=(animal$ NEAR/3 therap*) OR AK=(animal$ NEAR/3 therap*)

Date run: Fri 11 Oct 2024, 21:07:14 (GMT+0200)

Results: 8,393

9. TI=("natural environment$") OR AB=("natural environment$") OR AK=("natural environment$")

Date run: Fri 11 Oct 2024, 21:07:15 (GMT+0200)

Results: 41,654

10. TI=("nature experience$") OR AB=("nature experience$") OR AK=("nature experience$")

Date run: Fri 11 Oct 2024, 21:07:15 (GMT+0200)

Results: 578

11. TI=("forest bathing") OR AB=("forest bathing") OR AK=("forest bathing")

Date run: Fri 11 Oct 2024, 21:07:16 (GMT+0200)

Results: 216

12. TI=("shinrin yoku") OR AB=("shinrin yoku") OR AK=("shinrin yoku")
   Date run: Fri 11 Oct 2024, 21:07:16 (GMT+0200)
   Results: 102

13. TI=(ecotherap*) OR AB=(ecotherap*) OR AK=(ecotherap*)
   Date run: Fri 11 Oct 2024, 21:07:17 (GMT+0200)
   Results: 75

14. TI=(care farm*) OR AB=(care farm*) OR AK=(care farm*)
   Date run: Fri 11 Oct 2024, 21:07:17 (GMT+0200)
   Results: 5,352

15. TI=(therap* NEAR/4 farm*) OR AB=(therap* NEAR/4 farm*) OR AK=(therap* NEAR/4 farm*)
   Date run: Fri 11 Oct 2024, 21:07:17 (GMT+0200)
   Results: 392

16. TI=("therapeutic space$") OR AB=("therapeutic space$") OR AK=("therapeutic space$")
   Date run: Fri 11 Oct 2024, 21:07:18 (GMT+0200)
   Results: 345

17. #1 OR #2 OR #3 OR #4 OR #5 OR #6 OR #7 OR #8 OR #9 OR #10 OR #11 OR #12 OR #13 OR #14 OR #15 OR #16
   Date run: Fri 11 Oct 2024, 21:07:19 (GMT+0200)
   Results: 107,698

18. TI=("emotional disorder$") OR AB=("emotional disorder$") OR AK=("emotional disorder$")
   Date run: Fri 11 Oct 2024, 21:07:19 (GMT+0200)
   Results: 4,585

19. TI=("psychological disorder$") OR AB=("psychological disorder$") OR AK=("psychological disorder$")
   Date run: Fri 11 Oct 2024, 21:07:19 (GMT+0200)
   Results: 6,453

20. TI=("psychiatric condition$") OR AB=("psychiatric condition$") OR AK=("psychiatric condition$")

Date run: Fri 11 Oct 2024, 21:07:20 (GMT+0200)

Results: 7,919

21. TI=("depressive disorder$") OR AB=("depressive disorder$") OR AK=("depressive disorder$")

Date run: Fri 11 Oct 2024, 21:07:20 (GMT+0200)

Results: 57,812

22. TI=("schizophrenia spectrum disorder$") OR AB=("schizophrenia spectrum disorder$") OR AK=("schizophrenia spectrum disorder$")

Date run: Fri 11 Oct 2024, 21:07:21 (GMT+0200)

Results: 4,524

23. TI=("personality disorder$") OR AB=("personality disorder$") OR AK=("personality disorder$")

Date run: Fri 11 Oct 2024, 21:07:21 (GMT+0200)

Results: 30,234

24. TI=(psychosis) OR AB=(psychosis) OR AK=(psychosis)

Date run: Fri 11 Oct 2024, 21:07:22 (GMT+0200)

Results: 69,824

25. TI=("anxiety disorder$") OR AB=("anxiety disorder$") OR AK=("anxiety disorder$")

Date run: Fri 11 Oct 2024, 21:07:22 (GMT+0200)

Results: 49,730

26. TI=("affective disorder$") OR AB=("affective disorder$") OR AK=("affective disorder$")

Date run: Fri 11 Oct 2024, 21:07:23 (GMT+0200)

Results: 21,275

27. TI=("mood disorder$") OR AB=("mood disorder$") OR AK=("mood disorder$")

Date run: Fri 11 Oct 2024, 21:07:23 (GMT+0200)

Results: 27,063

28. TI=("psychiatric disorder$") OR AB=("psychiatric disorder$") OR AK=("psychiatric disorder$")

Date run: Fri 11 Oct 2024, 21:07:24 (GMT+0200)

Results: 56,738

29. TI=("psychiatric illness") OR AB=("psychiatric illness") OR AK=("psychiatric illness")

Date run: Fri 11 Oct 2024, 21:07:24 (GMT+0200)

Results: 8,017

30. TI=(mental NEAR/2 disorder$) OR AB=(mental NEAR/2 disorder$) OR AK=(mental NEAR/2 disorder$)

Date run: Fri 11 Oct 2024, 21:07:24 (GMT+0200)

Results: 74,135

31. #18 OR #19 OR #20 OR #21 OR #22 OR #23 OR #24 OR #25 OR #26 OR #27 OR #28 OR #29 OR #30

Date run: Fri 11 Oct 2024, 21:07:25 (GMT+0200)

Results: 346,668

32. TI=("occupational therap*") OR AB=("occupational therap*") OR AK=("occupational therap*")

Date run: Fri 11 Oct 2024, 21:07:26 (GMT+0200)

Results: 22,007

33. TI=("restorative therap*") OR AB=("restorative therap*") OR AK=("restorative therap*")

Date run: Fri 11 Oct 2024, 21:07:26 (GMT+0200)

Results: 483

34. TI=(neurorehabilitat*) OR AB=(neurorehabilitat*) OR AK=(neurorehabilitat*)

Date run: Fri 11 Oct 2024, 21:07:27 (GMT+0200)

Results: 6,273

35. TI=(reintegration$) OR AB=(reintegration$) OR AK=(reintegration$)

Date run: Fri 11 Oct 2024, 21:07:27 (GMT+0200)

Results: 7,620

36. TI=(recover*) OR AB=(recover*) OR AK=(recover*)
Date run: Fri 11 Oct 2024, 21:07:28 (GMT+0200)
Results: 1,531,942

37. TI=(rehabilitat*) OR AB=(rehabilitat*) OR AK=(rehabilitat*)
Date run: Fri 11 Oct 2024, 21:07:28 (GMT+0200)
Results: 291,664

38. #32 OR #33 OR #34 OR #35 OR #36 OR #37

PRISMA 2020 flow diagram for updated systematic reviews which included searches of databases, registers and other sources

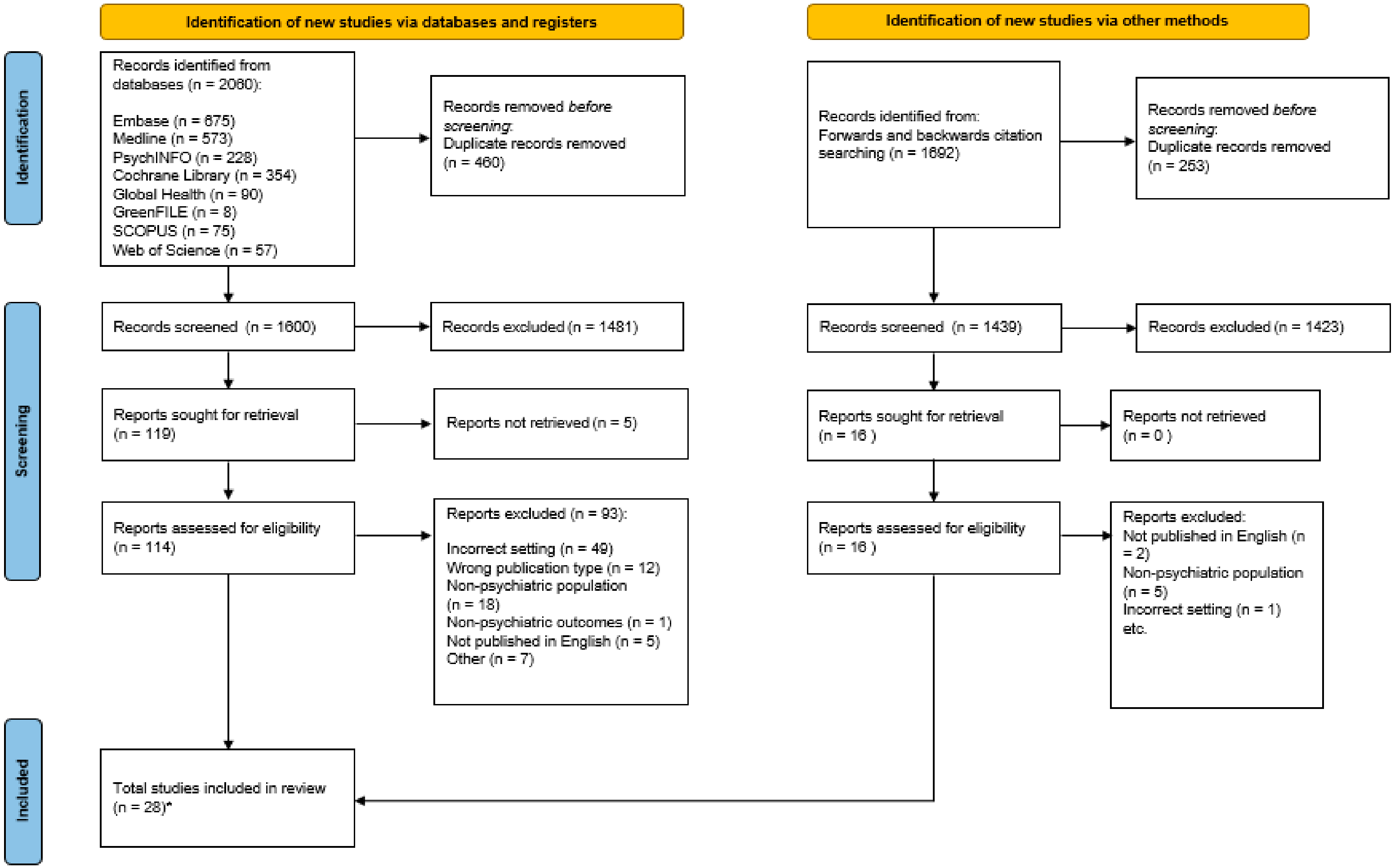

***Supplementary Figure S1** PRISMA 2020 flow diagram for study selection*

File S2. Intervention and comparator details

Supplementary Table S1 Intervention and control group details of the examined studies. NBI = nature-based intervention; NA = not applicable; NR = not reported; EHT = edible horticulture therap; NBR = nature-based rehabilitation.

Author(s)	Intervention Group Characteristics						Control Group Characteristics			
	NBI description	Time points & timing	n	Age M(SD)	Gender (M/F/O)	Criteria	Condition	n	Age M(SD)	Gender (M/F/O)
**Atta et al[35].**	Horticultural therapy using aromatic plants; included planting, propagation, sensory exercises, and reflection.	**T1** baseline; **T2** post-intervention; **T3** 3-month follow-up<br><br>Assessment window: as stated	60	27.28 (7.19)	NR	Same hospital; same inclusion	TAU	60	27.8 (7.55)	NR
**He et al[44].**	Horticultural therapy focused on five-sense activities (planting, watering, tasting, harvesting) using seasonal plants (lettuce, mint).	**T1** baseline; **T2** post-intervention<br><br>Assessment window: NR	30	44.20 (13.695)	Female	NA	TAU	29	46.66 (13.40)	Female
**Sisman et al[31].**	6-week plant cultivation (6 sessions: theoretical, practical gardening, and reflection), led by nurses at CMHC; each participant grew own plant.	**T1** baseline; **T2** post-intervention<br><br>Assessment window: as stated	40	40.05 (10.14)	23/17	Schizophre nia; same CMHC inclusion	TAU	40	42.35 (8.11)	25/15
**Oh et al[31,42].**	Horticultural therapy, involving planting, watering, fertilising, and harvesting.	**T1** baseline; **T2** post-intervention<br><br>Assessment window: NR	15	42.1 (13.0)	14/1	NA	**Active control:** leisure/exercise (e.g., discussions, stretching, ball games)	13	33.4 (9.4)	6/7
**Zhu et al[37].**	Horticultural programme led by therapists; indoor/outdoor gardening and cooking, with personal 0.5 m² plots.	**T1** baseline; **T2** week 4; **T3** week 12 (post)<br><br>Assessment window: Fixed	55	NR	24/21	NA	NA	55	NR	24/21

**Währborg et al[39].**	multimodal garden rehabilitation led by horticultural and clinical professionals, combining gardening, walking, relaxation, and psychotherapy.	**T1** 1-year pre; **T2** 1-year post<br><br>Assessment window: fixed	103	NR	11/92	Contemporaneous illness episode.	TAU	678	NR	78/600
**Pedersen et al[32]**	12-week farm animal-assisted "Green care" on dairy farms: work and interaction with dairy cattle (grooming, mucking, feeding, caring for calves, milking / physical contact) twice weekly alongside usual treatment.	**T1** baseline; **T2** week 4; **T3** week 8; **T4** week 12 (post); **T5** 3-month follow-up<br><br>Assessment window: fixed	16	40.5 (10.7)	5/11	Same inclusion	TAU	13	34.0 (6.6)	1/12
**Pedersen et al[41]**	Animal-assisted therapy involving farm work and animal contact	**T1** baseline; **T2** post-intervention<br><br>Assessment window: NR	14	37.4 (NR)	3/11	NA	NA	NA	NA	NA
**Berget et al[59]**	farm-based therapy (2×/week, 3 hr) involving animal care alongside standard therapy and medication.	**T1** baseline; **T2** post<br><br>Assessment window: videos: early (weeks 1–2) & late (weeks 11–12)	60	NR	NR	NA	TAU	30	NR	NR
**Shimizu et al[40]**	One-day sheep-care activity (feeding, herding, cleaning pens) with both intervention and control conditions attended by same participants.	**T1** 10:00–10:30; **T2** 15:00–15:30<br><br>Assessment window: Same day	14	56 (13.79)	9/5	Within-subjects	Same participants completed control session (standard facility routine)	14	56 (13.79)	9/5
**Walter et al[38]**	Surf Therapy (beach-based) and Hike Therapy (varied sites); optional pre-session yoga.	**T1** baseline; **T2** post-intervention; **T3** 3-month follow-up<br><br>Assessment window: T1 ≤2 weeks pre; T2 ≤2 weeks post	48	29.3 (6.2)	26/22	NA	**Active control:** hike therapy	48	27.0 (4.8)	20/28

**Keenan et al[33]**	Group walks (nature vs. urban routes), led by researcher and volunteers; identical structure across conditions.	**T1** baseline; **T2** post; **T3** 6-wk follow-up<br><br>Assessment timing: fixed	25	34.24 (9.63)	NR	NA	**Active control:** Urban walks	25	46.44 (12.50)	NR
**Barton et al[34]**	“green exercise” walking in local green spaces	**T1** baseline; **T2** post-intervention<br><br>Assessment window: as stated	24	43.4 (12.2)	12/12	Adults in Mind services	**Active controls:** indoor swimming or social club (no nature)	29	Swimming: 63.2 (11.5)<br><br>Social activities: 60.0 (14.1)	8/21
**Müller et al[43]**	3–4-week Greencare programme with weekly outdoor sessions integrating mindfulness, relaxation, and sensory engagement in nature.	**T1** admission**; T2** discharge**; T3** 3-month follow-up<br><br>Assessment window: fixed (per stay)	116	52.3 (NR)	45/71	Same clinical population	TAU/waitlist	111	51 (NR)	49/61
**Trkulja et al[57]**	Nature-Based Rehabilitation Program in Belgrade Botanical Garden	**T1** baseline**; T2** post<br><br>Assessment window: after 5 weeks	15	NR	NR	Psychiatric outpatients meeting same diagnostic criteria	**Active control:** indoor occupational/art therapy (no nature)	12	NR	NR
**Siu et al[58]**	Standardized Horticultural Therapy program	**T1** baseline**; T2** post-intervention**; T3** 3-month follow-up<br><br>Assessment window: T1/T2 within 1 week of start/end	41	50.8 (10.5)	20/21	Attending usual vocational rehabilitati on programs; not participatin g in structured HT	TAU	41	49.7 (8.7)	17/24
**Kam & Siu[56]**	Standardised horticultural activity programme delivered by a registered OT; five outdoor themed gardens	**T1** baseline; **T2** post (end of 2-wk programme)<br><br>Assessment timing: fixed	12	45.3 (10.38)	8/4	Same eligibility	**Active control:** Conventional sheltered workshop training	12	43.3 (11.7)	9/3
**Iwata et al[56]**	forest therapy co-led by clinical and forestry staff.	PANAS: week 8 & week 13; HDRS/BDI: pre & post (subset); interviews: 1 wk post	15	47 (NR)	3/12	NA	NA	NA	NA	NA

		Assessment timing: fixed (per item)							
**Pálsdóttir et al[47]**	multimodal garden rehabilitation combining horticultural, psycho-occupational, and physiotherapy in greenhouses/gardens.	**T1** post-intervention interviews<br><br>Assessment window: within 1 month post	59	45.5 (NR)	9/50	NA	NA	NA	NA	NA
**Wästberg et al[46]**	group garden therapy (2×/week, 3 hr) including gardening, creative, and mindfulness activities with reflective discussions.	Interview 1 (weeks 5–9); Interview 2 (~12 weeks post)<br><br>Assessment window: as stated	8	NR	1/7	NA	NA	NA	NA	NA
**Cerwén et al[52]**	NBR in a therapeutic garden at the university campus; group sessions combining restoration, sensory engagement, and cultivation tasks.	Post-intervention interviews<br><br>Assessment timing: NR	59	NR	9/50	NA	NA	NA	NA	NA
**Barley et al[53]**	Weekly nature and arts sessions at Sydenham Garden, combining gardening and art in a user-led community setting.	NR	16	NR	9/7	NA	NA	NA	NA	NA
**Fieldhouse[50]**	Weekly horticultural allotment sessions; long-term membership	NR	9	46.0 (13.53)	6/3	NA	NA	NA	NA	NA
**Joung et al[54]**	Care-farming programme (sowing, harvesting, art, barefoot walking, mindfulness, and closing celebration).	During programme (observations); post-programme (focus groups)<br><br>Assessment timing: as stated	6	39.0 (11.0)	2/4	NA	NA	NA	NA	NA

**Iancu et al[49]**	Daily work-based activities on care farms (gardening, feeding animals, cleaning barns, kitchen tasks, tool repair) under farmer supervision.	NR	14	39.6 (13.3)	9/5	NA	**Active control:** Work and creative projects	12	45.9 (8.5)	7/5
**Pedersen et al[36]**	Dairy-farm programme involving routine animal care (feeding, milking, cleaning) and physical contact with cattle.	**T1** post-intervention interviews<br><br>Assessment window: 0–14 months post	8	37.62 (11.58)	1/7	NA	NA	NA	NA	NA
**Leighton et al[48]**	Experiential psychotherapy at inpatient facility; group activities to build awareness, trust, and reflection.	**T1** during workshop**; T2** immediate post focus group; **T3** ≤2 w post interviews<br><br>Assessment timing: fixed for T1/T2; T3 ≤2 window	6	NR	3/3	NA	NA	NA	NA	NA
**Cooley et al[51]**	Fortnightly 2-hr "Walk to Wellbeing" sessions (Feb–Dec): urban or nature walks (~60–90 min) followed by café reflection, facilitated by OTs.	Interviews/diaries<br><br>Assessment window: NR	7 patients + 22 historica l	52.14 (6.20)	15/14	NA	NA	NA	NA	NA

File S3. Quantitative outcome tables

Supplementary Table S3. Quality of life and wellbeing outcomes of the examined studies.

Author(s)	Outcome Tool	Subscale / Measure	Group	Statistical Test	Pre Mean (SD)	Post Mean (SD)	p-value
**He et al.**	Life Satisfaction Index A (LSIA)	Total	IG / CG	Paired t-test	IG: 22.07 (7.39); CG: 19.56 (7.00)	IG: 23.29 (7.57); CG: 16.22 (5.77)	0.000
**Berget et al**	Quality of Life Scale – Norwegian version (QOLS-N)	Total	IG / CG	ANOVA	IG: 64.3 (14.93); CG: 63.2 (14.06)	After intervention – IG: 64.3 (17.09), CG: 64.4 (13.52); 6-month FU – IG: 66.7 (16.86), CG: 66.0 (15.25)	After intervention > 0.05; 6-month FU vs pre > 0.05
**Müller et al.**	Self-Compassion Scale – Short Form (SSCS-S)	Total	IG / CG	Linear mixed models for repeated measures	IG: 2.74 (NR); CG: 2.74 (NR)	IG: 3.25 (NR); CG: 3.01 (NR)	0.004
	Mindfulness and Awareness Scale (MAAS)	Total	IG / CG	Linear mixed models for	IG: 3.57 (NR); CG: 3.60 (NR)	IG: 4.29 (NR); CG: 4.11 (NR)	0.154

				repeated measures			
**Siu et al.**	Chinese version of the Short Warwick-Edinburgh Mental Well-being Scale (C-SWEMWBS)	Mental well-being	IG / CG	ANCOVA	IG: 3.10 (0.66); CG: 3.18 (0.71)	IG: 3.30 (0.73); CG: 2.95 (0.70)	0.01
**Keenan et al.**	Warwick-Edinburgh Mental Well-being Scale (WEMWBS)	Total	IG / CG	t-test	IG: 44.80 (11.77); CG: 43.96 (12.50)	Post: IG 53.32 (9.35), CG 49.80 (10.65); Follow-up: IG 63.44 (3.65), CG 42.68 (3.65)	Post: NS (NR); Follow-up: < .05
	Connectedness to Nature Scale (CNS)	Total	IG / CG	t-test	IG: 3.12 (0.33); CG: 3.20 (0.24)	Post: IG 3.86 (0.28), CG 3.40 (0.25); Follow-up: IG 4.05 (0.21), CG 3.34 (0.34)	< .05
**Kam et al.**	Personal Well-being Index – Chinese version	Total	IG / CG	t-test	IG: 49.5 (11.8); CG: 53.2 (14.9)	IG: 0.60 (14.21); CG: 1.50 (6.07)	0.84
**Atta et al.**	Herth Hope Index (HHI)	Total	IG / CG	Independent samples t-test	IG: 19.25 (3.41); CG: 18.05 (3.62)	IG: 25.60 (6.30); CG: 18.70 (3.80)	< 0.001

	Ryff Psychological Wellbeing Scale (PWBS)	Total	IG / CG	Independent samples t-test	IG: 43.28 (9.10); CG: 45.43 (9.45)	IG: 63.23 (8.37); CG: 43.65 (8.93)	< 0.001

Supplementary Table S5. Psychosocial functioning outcomes of the examined studies.

Author(s)	Outcome tool	Subscale / measure	Group	Statistical test	Pre Mean (SD)	Post Mean (SD)	p-value
**Pedersen et al**	Generalized Self-Efficacy Scale (GSE)	Total	IG	Spearman's rho (pre–post change)	22.3 (1.6)	25.6 (2.1)	Mucking: p = 0.01; Milking: p = 0.08
**Berget et al**	Generalized Self-Efficacy Scale (GSE)	Total	IG / CG	ANOVA; independent t-test	IG: 23.1 (5.12); CG: 25.6 (6.40)	After intervention: IG 23.5 (6.56), CG 25.3 (6.62); 6-month follow-up: IG 25.7 (5.93), CG 25.4 (5.92)	After interventio n: p > 0.05; 6-month follow-up vs pre: p = 0.05; 6-month

							follow-up vs post: p = 0.02
	Coping Strategies Scale of the Pressure Management Indicator	Total	IG / CG	ANOVA; independent t-test; within-IG t-test	IG: 31.6 (8.51); CG: 32.2 (7.38)	After intervention: IG 32.8 (8.67), CG 31.4 (8.69); 6-month follow-up: IG 34.3 (8.10), CG 31.6 (8.02)	Between-group: all comparison s > 0.05 (NS, α = 0.05); within IG, 6-month follow-up vs pre: p = 0.03; CG change tests NR
**Siu et al.**	Social Exchange and Support Measure (SESM)	Experiential knowledge provided; Emotional support provided; Experiential knowledge received; Emotional support	IG	Paired t-test	Experiential provided: 2.98 (1.73); Emotional provided: 3.63 (1.70); Experiential received: 3.15 (1.63); Emotional	Experiential provided: 2.70 (1.72); Emotional provided: 3.48 (1.63); Experiential received: 3.29 (1.48); Emotional received: 3.95 (1.59);	Experientia l provided: p = 0.27; Emotional provided: p = 0.49; Experientia

		received; Humour exchanged; Unwanted behaviour received			received: 3.61 (1.70); Humour: 2.32 (1.64); Unwanted: 1.52 (1.13)	Humour: 2.28 (1.20); Unwanted: 1.54 (0.94)	l received: p = 0.60; Emotional received: p = 0.13; Humour: p = 0.90; Unwanted: p = 0.92
**Pedersen et al**	Generalized Self-Efficacy Scale (GSE)	Total	IG / CG	Paired t-test	IG: 22.8 (5.3); CG: 19.8 (6.2)	IG: 25.6 (6.7); CG: 21.5 (6.6)	IG: p = 0.045; CG: NS (exact p NR, > 0.05)
**Barton et al.**	Rosenberg Self-Esteem Scale (RSE)	Total	Green Exercise	Paired t-test	23.6 (6.0)	21.0 (6.1)	p < 0.0001*
**Barton et al.**	Rosenberg Self-Esteem Scale (RSE)	Total	Swimmin g	Paired t-test	24.1 (5.0)	22.8 (4.9)	p < 0.0001*
**Barton et al.**	Rosenberg Self-Esteem Scale (RSE)	Total	Social Activities	Paired t-test	20.7 (6.2)		

Supplementary Table S3. Functional outcomes of the examined studies.

Author(s)	Outcome Tool	Subscale / Measure	Group	Statistical Test	Pre Mean (SD)	Post Mean (SD)	p-value
**He et al.**	Scale of Social Functioning for Psychotic Inpatients (SSFPI)	Total	IG / CG	Paired t-test	IG: 28.37 (6.54); CG: 26.17 (5.47)	IG: 34.87 (5.97); CG: 26.07 (5.23)	0.000
**Siu et al.**	Engagement in Meaningful Activities Survey (EMAS)	Engagement in meaningful activity	IG / CG	ANCOVA	IG: 2.72 (0.62); CG: 2.58 (0.69)	IG: 2.74 (0.69); CG: 2.40 (0.74)	0.06
	Affect and Engagement Checklist (AEC)	Affect	IG / CG	ANCOVA	IG: 0.035 (1.15); CG: −0.29 (0.72)	IG: 0.40 (1.07); CG: 0.22 (1.00)	0.31
	Affect and Engagement Checklist (AEC)	Engagement	IG / CG	ANCOVA	IG: 97.06% (5.67); CG: 83.33% (21.91)	IG: 96.16% (7.06); CG: 81.00% (18.14)	0.001
**Kam et al.**	Work Behavioral Assessment	Work Habit (WH)	IG / CG	t-test	IG: 7.2 (1.5); CG: 7.7 (2.1)	IG: 0.10 (0.32); CG: 0.16 (0.72)	0.79
	Work Behavioral Assessment	General Work Behavior (GW)	IG / CG	t-test	IG: 24.2 (6.2); CG: 28.0 (8.5)	IG: 2.70 (3.06); CG: 0.92 (1.17)	0.08
	Work Behavioral Assessment	Work-related Social and Emotional Behavior (WS)	IG / CG	t-test	IG: 28.5 (5.3); CG: 30.3 (4.8)	IG: 0.90 (2.18); CG: 0.42 (0.90)	0.49
	Work Behavioral Assessment	Total	IG / CG	t-test	IG: 59.9 (11.1); CG: 65.9 (14.6)	IG: 3.70 (4.42); CG: 1.50 (2.28)	0.15

**Atta et al.**	Modified Social Adjustment Scale (MSAS / SAS-SR)	Total	IG / CG	Independent samples t-test	IG: 62.12 (13.03); CG: 64.87 (13.64)	IG: 66.00 (13.26); CG: 116.35 (20.69)	< 0.001
**Sisman et al.**	Functional Remission of General Schizophrenia Scale (FROGS)	Total	IG / CG	Wilcoxon signed-rank test	IG: 55.42 (10.97); CG: 53.10 (10.51)	IG: 62.67 (8.75); CG: 51.90 (7.09)	IG: < 0.001; CG: 0.07 (NS)

Supplementary Table S2 Physical benefits of the examined study.

Author(s)	Outcome tool	Subscale / measure	Group	Statistical test	Pre Mean (SD)	Post Mean (SD)	p-value
**Shimizu et al**	Cortisol	N/A	IG / CG	Mann–Whitney U test	IG: 0.238 (0.035); CG: 0.260 (0.032)	IG: 0.199 (0.027); CG: 0.173 (0.011)	IG: 0.199
	Testosterone	N/A	IG / CG	Mann–Whitney U test	IG: 0.146 (0.011); CG: 0.154 (0.007)	IG: 0.224 (0.076); CG: 0.134 (0.007)	IG: 0.003

Supplementary Table S6 Illustrates each theme and subtheme alongside exemplar quotes, the source study, and the corresponding in-text reference code (S1–S26)

Theme and subtheme	Quotes	Study	In text reference
**Being in Nature**			
**Experiences of the Outdoors**	*"When you are outside, and you get that fresh air in your lungs. It's hard to explain but it just brightens you up and puts a smile on your face… when I am out there, I smile more."*	Leighton et al[48]	S1
	"It's a really good feeling ... away from the traffic and the noise. I think it makes a lot of people feel better."	Cooley et al[51]	S2
	"I remember our horticulture class was suspended due to typhoon that day, but the plants grow even bigger and stronger after the severe storm. It is pleasurable to see the shoots growing into plants. Plants are amazing. Some plants and flowers will close at night, but re-open in the morning. I did not realize that plants are so resilient."	Siu et al[58]	S3
**Sensory Aspects**	*"I was so aware of how the grass smelled and I had also started to feel like a little more alive there."*	Pálsdóttir et al[47]	S4
	"But you know that feeling of it just being you and nature and it's completely quiet, what, you hear a stream trickle somewhere and you hear a bird that... you hear some slight rustling in the trees or whatever, just wonderful and it's so incredibly beautiful that you just...".	Cerwén et al[52]	S5
	"It's a difference in texture and colour. That's dark – you know that you pick it up it's gonna break in your hands – very soft; and that's grey – and that's very dry and brittle. I'm not a gardener but I like the smell of that soil too ... 'cos, I mean, you can't get more real than digging up a clod of earth, and the smell of that – it's real."	Fieldhouse[50]	S6
**Personal Growth**			
**Pathways of Change**	*"I can also say: I stop with [going to] that farm. But then again I have nothing. What should I do then? Because then I lie in bed the whole day ... That's not a solution. So I simply pushed myself."*	Iancu et al[49]	S7
	"You feel a sense of acceptance, people aren't judging you ... we don't really know what different people's situations are, but people, you know, are accepted whatever, and that's lovely."	Barley et al[53]	S8
	"I'm not thinking about drugs or anything like that while I'm down there ... I'm thinking about how I can live my – a better life."	Fieldhouse[50]	S9
**Professional Opportunities**	*"Horticulture could be a kind of play, but it also could be work. After learning horticulture, I have more and more plants at home, such as tomatoes, cactus, etc. I see people working in gardens or parks, they must know about horticulture. I*	Siu et al[58]	S10

	am interested in this kind of work. It may be good for me to open a flora store. Maybe we could run a stall at the annual Flower Market."		
	"One participant mentioned that he wanted to pursue a career in horticulture, while most of the other participants stated they would like to further engage in horticultural activities as a hobby."	Siu et al[58]	S11
	"It motivated me to go to the interview and take on the job"	Cooley et al[51]	S12
**Psychological Well-Being**			
**Emotional Well-being**	*"[the farmer] can express everything so nice, pays attention only to the good things, everything that's negative you can leave behind you, or ignore it, lets it slide away ... It works perfectly ... you start to copy such an attitude automatically."*	Iancu et al[49]	S13
	"First I went to the calf barn and cuddled with the calves, and then my mood was elevated."	Pedersen et al[36]	S14
	"It feels like your stresses are kind of moving away and… there's no need to be anxious, there's no need to worry about things, you can just let go."	Leighton et al[48]	S15
**Self-Identity**	*"The farmer said it was so easy to work together with me, because I understood what to do, and then I did it. So we worked very well together."*	Pedersen et al[36]	S16
	"Sam felt as if she was getting to know herself, realising that she knew very little of whom she really was."	Wastberg et al[46]	S17
	"The connectedness to nature through olfactory stimuli was expressed as reconnecting with the inner self."	Pálsdóttir et al[47]	S18
**Cognitive Outcomes**	*"I become more agile, I use my brain more."*	Siu et al[58]	S19
	"I have become more attentive and aware. It must be connected to the possibility to ill my day with something different from the "internal" work."	Pedersen et al[36]	S20
	"Most participants described *'a clearer head'* or were aware of *'thinking differently'* on the allotment. They noted improved concentration and flow experiences."	Fieldhouse[50]	S21
**Social Relationships**	*"I was surprised to see that some of the activities that appeared to be simplistic in nature really got people talking."*	Leighton et al[48]	S22
	"When you're with other people you can try things out – can't you, really. 'Reality testing' I believe it's called ... I mean, you get some feedback to what you say, hopefully."	Fieldhouse[50]	S23
	"Just knowing I am not alone with what is going on here… allows me to connect."	Leighton et al[48]	S24

**Physical Benefits**	"Most of the participants appreciated the feeling of being tired after participating in physical activities and work. However, several participants emphasized that the work and especially the work with the animals also gave them energy."	Pedersen et al[36]	S25
	"He talked about the workshop as, *'physically freeing'*".	Leighton et al[48]	S26

Quality Appraisal	Studies										
**CASP - Randomised Control Trial**	Berget et al. (2007)	Berget et al. (2008)	He et al. (2020)	Siu et al. (2020)	Walter et al. (2023)	Zhu et al. (2016)	Sisman et al. (2020)	Pedersen et al. (2015)	Kam & Siu (2010)	Atta et al. (2025)	Keenan et al. (2021)
**Did the study address a clearly formulated research question?**	Yes	Yes	Yes	Yes	Yes	Yes	Yes	Yes	Yes	Yes	Yes
**Was the assignment of participants to interventions randomised?**	Yes	Yes	Yes	Yes	Yes	Yes	Yes	Yes	Yes	Yes	Yes
**Were all participants who entered the study accounted for at its conclusion?**	No	No	No	No	No	No	Yes	No	No	No	Can't Tell
**Were the participants 'blind' to intervention they were given?**	No	No	No	No	No	No	No	No	No	No	No
**Were the investigators 'blind' to the intervention they were giving to participants?**	No	No	No	No	No	Yes	No	No	No	No	Can't Tell
**Were the people assessing/analysing outcome/s 'blinded'?**	No	No	Yes	Yes	Yes	Yes	No	Can't Tell	Yes	Can't Tell	Can't Tell

**Were the study groups similar at the start of the randomised controlled trial?**	No	No	Yes	Yes	No	Yes	Yes	No	Yes	Yes	Yes
**Apart from the experimental intervention, did each study group receive the same level of care (that is, were they treated equally)?**	No	Yes	Can't Tell	Can't Tell	No	Yes	Can't Tell	No	Can't Tell	Yes	Yes
**Were the effects of intervention reported comprehensively?**	Yes	Yes	Yes	Yes	Yes	Yes	Yes	Yes	Yes	Yes	Yes
**Was the precision of the estimate of the intervention or treatment effect reported?**	Yes	Yes	Yes	No	Yes	Yes	Yes	No	No	No	No
**Do the benefits of the experimental intervention outweigh the harms and costs?**	Can't Tell	Can't Tell	Can't Tell	Can't Tell	Can't Tell	Yes	Can't Tell	Can't Tell	Can't Tell	Can't Tell	Can't Tell
**Can the results be applied to your local population/in your context?**	Yes	Yes	Yes	Yes	No	Yes	Yes	Can't Tell	Yes	Yes	Yes

**Would the experimental intervention provide greater value to the people in your care than any of the existing interventions?**	Can't Tell	Can't Tell	Can't Tell	Can't Tell	Can't Tell	Can't Tell	Can't Tell	Can't Tell	Can't Tell	Can't Tell	Can't Tell
**CASP Cohort Studies**	Währborg et al. 2014	Trkulja et al. (2021)	Shimizu et al. (2023)	Pederson et al. (2011)	Oh et al. (2018)	Muller et al. (2025)	Barton et al. (2012)	Iwata et al. (2016)			
**Did the study address a clearly focused issue?**	Yes	Yes	Yes	Yes	Yes	Yes	Yes	Yes			
**Was the cohort recruited in an acceptable way?**	Yes	Can't Tell	No	No	No	Can't Tell	Yes	Yes			
**Was the exposure accurately measured to minimise bias?**	Can't Tell	Yes	No	Can't Tell	Yes	Yes	Yes	Yes			
**Was the outcome accurately measured to minimise bias?**	Yes	No	No	Yes	Yes	Yes	Yes	Yes			
**Have the authors identified all important confounding factors?**	No	No	No	Yes	No	Yes	Yes	Yes			
**Have they taken account of the confounding factors in the design and/or analysis?**	Yes	No	No	Yes	No	Yes	Yes	Yes			

**Was the follow up of subjects complete enough?**	Can't Tell	Yes	No	Yes	Yes	Yes	No	Can't Tell			
**Was the follow up of subjects long enough?**	Yes	No	No	Yes	No	Yes	No	No			
**What are the results of this study?**	Yes	Yes	Yes	Yes	Yes	Yes	Yes	Yes			
**How precise are the results?**	Yes	Yes	Can't Tell	Yes	Yes	Yes	Can't Tell	Can't Tell			
**Do you believe the results?**	Yes	Yes	Can't Tell	Yes	Yes	Yes	Yes	Yes			
**Can the results be applied to the local population?**	Can't Tell	Can't Tell	No	Can't Tell	Can't Tell	Can't Tell	Yes	Yes			
**Do the results of this study fit with other available evidence?**	Yes	Yes	Yes	Yes	Yes	Yes	Yes	Yes			
**What are the implications of this study for practice?**	Can't Tell	Yes	Can't Tell	Yes	Yes	Yes	Yes	Yes			
**Casp Qualitative Research**	Cerwen et al. (2016)	Cooley et al. (2020)	Fieldhouse (2003)	Howarth et al. (2018)	Iancu et al. (2013)	Leighton et al. (2021)	Palsdottir et al. (2021)	Pederson et al. (2012)	Wasterberg et al. (2020)	Barley et al. (2012)	Joung et al. (2025)
**Was there a clear statement of the aims of the research?**	Yes	Yes	Yes	Yes	Yes	Yes	Yes	Yes	Yes	Yes	Yes

**Is a qualitative methodology appropriate?**	Yes	Yes	Yes	Yes	Yes	Yes	Yes	Yes	Yes	Yes	Yes
**Was the research design appropriate to address the aims of the research?**	Yes	Yes	Yes	Yes	Yes	Yes	Yes	Yes	Yes	Yes	Yes
**Was the recruitment strategy appropriate to the aims of the research?**	Yes	Yes	Yes	Yes	Yes	Yes	Yes	Can't Tell	Yes	Yes	Yes
**Was the data collected in a way that addressed the research issue?**	Yes	Yes	Can't Tell	Yes	Yes	Yes	Yes	Can't Tell	Yes	Yes	Yes
**Has the relationship between researcher and participants been adequately considered?**	No	No	Can't Tell	No	No	No	No	Yes	Yes	No	Yes
**Have ethical issues been taken into consideration?**	Yes	Yes	Yes	Yes	Yes	Yes	Yes	Yes	Yes	Yes	Yes
**Was the data analysis sufficiently rigorous?**	Yes	Yes	Yes	Yes	Yes	Yes	Yes	Yes	Yes	Yes	Yes
**Is there a clear statement of findings?**	Yes	Yes	Yes	Yes	Yes	Yes	Yes	Yes	Yes	Yes	Yes
**How valuable is the research?**	Yes	Yes	Yes	Yes	Yes	Yes	Yes	Yes	Yes	Yes	Yes
